# Optimisation of Pharmaceutical Cocrystal Dissolution Performance through a Synergistic Precipitation Inhibition

**DOI:** 10.1007/s11095-023-03532-x

**Published:** 2023-05-15

**Authors:** Kejing Shi, Mingzhong Li

**Affiliations:** School of Pharmacy, De Montfort University, Leicester, LE1 9BH UK

**Keywords:** cocrystal, dissolution, flufenamic acid, polymers, precipitation, supersaturation

## Abstract

**Objectives:**

Polymeric excipients play an important role in a cocrystal formulation to act as precipitation inhibitors to maximize the potential. Otherwise, a stable form of the parent drug will be recrystallized on the dissolving cocrystal surface and/or in the bulk solution during the cocrystal dissolution process, negating the solubility advantage. The objectives of this work were to investigate the potential of using combined polymers to maximise the dissolution performance of surface precipitation pharmaceutical cocrystals.

**Methods:**

The dissolution performance of a highly soluble flufenamic acid and nicotinamide (FFA-NIC) cocrystal has been systematically studied with predissolved or powder mixed with a single polymer, including a surface precipitation inhibitor [i.e., copolymer of vinylpyrrolidone (60%) /vinyl acetate (40%) (PVP-VA)] and two bulk precipitation inhibitors [i.e., polyethylene glycol (PEG) and Soluplus (SLP)], or binary polymers combinations.

**Results:**

A single polymer of PVP-VA prevented the FFA surface precipitation for an enhanced dissolution performance of FFA-NIC cocrystal. Unfortunately, it cannot sustain the supersaturated FFA concentration in the bulk solution. A combination of two polymers of PVP-VA and SLP has shown a synergistic inhibition effect to enhance the dissolution advantage of FFA-NIC cocrystal.

**Conclusions:**

The dissolution of a cocrystal with surface precipitation of the parent drug can be described as: i) the cocrystal surface contacting the dissolution medium; ii) the cocrystal surface dissolving; iii) the parent drug precipitation on the dissolving surface; and iv) the parent drug particles redissolving. A combination of two types of polymers can be used to maximise the cocrystal performance in solution.

**Graphical Abstract:**

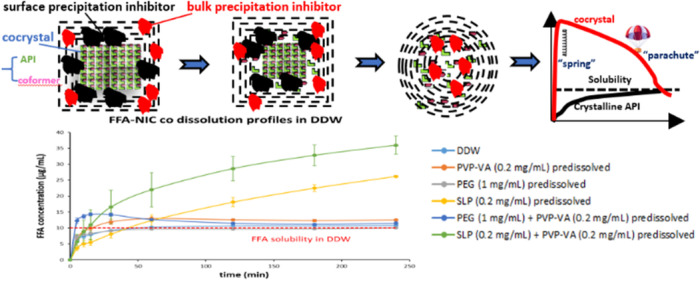

**Supplementary Information:**

The online version contains supplementary material available at 10.1007/s11095-023-03532-x.

## Introduction

Solid crystalline forms of active pharmaceutical ingredients (APIs) are preferred in the drug product development because they can be easily formulated into tablets and capsules. However, many APIs exhibit poor solubility and low dissolution rates in the aqueous environments of the gastrointestinal tract, leading to erratic dissolution performances and low oral bioavailability. Pharmaceutical cocrystallisation has become an attractive strategy for the discovery of new solid forms of an API with improved physicochemical properties, such as solubility, dissolution rate, stability and mechanical properties, through non-covalent interactions with a pharmaceutical cocrystal former molecule (called coformer) [1]. Successful applications of cocrystallisation to the pharmaceutical industry have brought several cocrystal drug products into the market, such as Suglat®, Entresto®, and Steglatro® [2].

Although the promising benefits, developing pharmaceutical cocrystal formulations is challenging because of the instability of pharmaceutical cocrystals in both solid and solution states. In the solid state, cocrystals could be susceptible to phase conversion at high humidity or in the presence of excipients in the formulation [3, 4]. In the solution state, due to a supersaturated state of the API concentration generated by the cocrystal dissolution, it is of critical importance to prevent the recrystallisation of the parent API. Otherwise, they could be recrystallised on the dissolving cocrystal surface (i.e., the surface precipitation mechanism) or in the bulk solution (i.e., the bulk precipitation mechanism), leading to the loss of an improved performance [5–10]. Many previous studies have shown that a polymeric excipient can be included in a cocrystal formulation to act as a precipitation inhibitor to maximise its potential [5, 11–13]. Polyvinylpyrrolidone (PVP) or the copolymer vinylpyrrolidone/vinyl acetate (PVP-VA) can act as a surface precipitation inhibitor in solution to enhance the dissolution performance of the cocrystal of flufenamic acid and nicotinamide (FFA-NIC) by preventing the surface precipitation of the parent drug. In contrast, polyethylene glycol (PEG) acted as a bulk precipitation inhibitor to improve the dissolution performance of the cocrystal of flufenamic acid and theophylline (FFA-TP) to prevent the parent drug precipitation in the bulk solution [5]. Selection of an effective polymeric inhibitor plays a key role to maximise the cocrystal dissolution performance in a formulation. An inappropriate inhibitor in the formulation could have an adverse effect on the cocrystal dissolution performance. For example, a surface precipitation inhibitor of PVP or PVP-VA actually reduced the dissolution performance of the cocrystal with bulk precipitation of the parent drug, FFA-TP, whilst the bulk precipitation inhibitor of PEG did not have any effect on the dissolution performance of the cocrystal with surface precipitation of the parent drug, FFA-NIC [5].

Generally, cocrystals, which cause surface precipitation of parent drugs, are of significantly improved solubility and dissolution rates [6, 14]. Therefore, when they were selected as lead candidates for drug development, surface precipitation inhibitors are needed in the formulations. Apart from preventing the surface precipitation of the parent drug during dissolution of the cocrystal, a bulk precipitation inhibition is also needed because a supersaturated state of the parent drug could be generated in the bulk solution. A recent study has indicated that a good surface precipitation inhibitor, such as PVP or PVP-VA, is not guaranteed to be an effective bulk solution precipitation inhibitor [15]. Therefore, a new formulation strategy of combining both surface precipitation and bulk precipitation inhibitors should be explored.

The specific objectives of this work were to investigate the potential of using combined polymers to maximise the dissolution performance of surface precipitation pharmaceutical cocrystals across different dissolution environments. This study firstly examined the dissolution performances of a cocrystal with surface precipitation of the parent drug (FFA-NIC) as the model cocrystal in double distilled water (DDW) in the absence and presence of an individual polymer, including a polymeric surface precipitation inhibitor, i.e., PVP-VA, and two different bulk precipitation inhibitors of PEG and Soluplus (SLP) [5, 6]. PVP-VA and PEG have been widely applied as crystallization inhibitors in various drug delivery systems [16, 17]. SLP, acting as a solubilizer, is a water-soluble graft copolymer consisting of PEG, polyvinyl acetate and polyvinyl caprolactam, showing an excellent solubilizing effect on biopharmaceutics classification system (BCS) class II substances [18, 19]. In recent years, the strategy to use SLP as a ternary substance with cyclodextrin has been proven to be effective to improve solubility, dissolution rates and bioavailability for drugs, such as FFA, itraconazole, tacrolimus, and docetaxel [20]. Subsequently, the synergistic effects of a combination of the predissolved surface and bulk precipitation inhibitors on the FFA-NIC cocrystal release profiles were examined. In comparison, the dissolution tests of the parent drug FFA and the cocrystal with bulk precipitation of the parent drug (FFA-TP) under the same environments were also conducted. The detailed chemical structures of the drug (FFA), coformers (i.e., NIC and TP) and polymers (i.e., PVP-VA, PEG and SLP) are shown in Table I.

**Table I:**
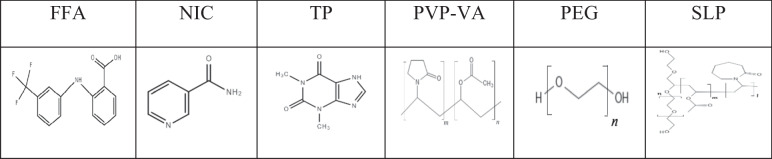
Chemical Structures of the Drug, Coformers and Monomer units of Polymers

In practice, polymers used in the formulation cannot be predissolved in the solution. Thus, in this work, the FFA release performances were examined in the powder mixtures of FFA-NIC cocrystal with an individual or combined polymeric excipients under non-sink (i.e. PBS pH 4.5) and sink (i.e., PBS pH 6.8) environments. Finally, insights into the dissolution and recrystallisation mechanisms of a cocrystal system were discussed.

## Materials and Methods

### Materials

Flufenamic acid form I (FFA I), nicotinamide (NIC) (≥ 99.5% purity), theophylline (TP) (≥ 99.5% purity), potassium dihydrogen phosphate (KH_2_PO_4_), and sodium hydroxide (NaOH) were purchased from Sigma- Aldrich (Dorset, UK). Copolymer of vinylpyrrolidone (60%) /vinyl acetate (40%) (PVP-VA, Plasdone™ S-630) was kindly donated by Ashland Inc. (Schaffhausen, Switzerland). Poly(ethylene glycol) (PEG) 4000 was purchased from Sigma-Aldrich (Dorset, UK). Soluplus® (SLP) was donated by BASF (Ludwigshafen, Germany). Methanol (HPLC grade) and acetonitrile (HPLC grade) were purchased from Fisher Scientific (Loughborough, UK) and used as received. Double distilled water (DDW) was generated from a bi-distiller (WSC044.MH3.7, Fistreem International Limited, Loughborough, UK) and used throughout the study.

### Methods

#### Phosphate Buffer Solution (PBS) Preparation

PBS pH 6.8 (0.01 M) and PBS pH 4.5 (0.01 M) as dissolution media were prepared according to British Pharmacopoeia 2018. For PBS pH 6.8, 50 mL of 0.2 M potassium dihydrogen phosphate and 22.4 mL of 0.2 M sodium hydroxide were mixed and diluted to 1000 mL with DDW. For PBS pH 4.5, 1.361 g of potassium phosphate monobasic dissolved in 1000 mL DDW. pH value of the solution was adjusted using sodium hydroxide solution if necessary.

#### Cocrystal Synthesis

The FFA-NIC and FFA-TP cocrystal powders were generated by cooling crystallisation using Polar Bear Plus Crystal (Cambridge Reactor Design Ltd, UK). A 1:1 molar ratio mixture of FFA and NIC or TP powders was added into the cosolvent (70% v/v acetonitrile: 30% v/v water) in a 20 mL vial and held at 45°C until all solids were dissolved. The temperature was then reduced to 0°C at a cooling rate of 0.3°C/min. The powders formed were isolated by paper filtration and air-dried. Prior to further usage, the obtained powders were analysed by PXRD for confirmation of the cocrystal formation by comparing the predicted PXRD pattern retrieved from the Cambridge Structural Database (CSD), i.e., CSD reference: ZIQDUA for FFA-TP or EXAQAW for FFA-NIC.

#### Equilibrium Solubility of FFA in DDW and PBS pH 4.5 in the Absence and Presence of a Predissolved Single Polymer or a Combination

The FFA solubility was determined by suspending an excess amount of crystalline materials of FFA I in small vials with 20 mL of a selected dissolution medium (i.e., DDW or PBS pH 4.5) in the absence and presence of a predissolved single polymer and a combination. The detailed polymers and their corresponding concentrations were 0.2 mg/mL of PVP-VA, 1 mg/mL of PEG and 0.2 mg/mL of SLP, which were based on the previous study [5] and the solubility curve obtained in the preliminary study in Figure S5 in the supporting materials. All of the suspensions were kept at 37 ± 0.5°C in a water bath with a shaking rate of 150 rpm for 24 h. Each of the supernatants was separated from excess solids in solution by centrifugation at 13,000 RPM for 1 min in an MSB 010.CX2.5 centrifuge (MSE Ltd., London, U.K.). Subsequently, the supernatant was diluted using ethanol, and the FFA concentration was determined using HPLC. The solid residues retrieved from the tests were dried for 24 h at ambient temperature and then analysed by PXRD. Each of the experiments was conducted in triplicate, and data were reported as an average concentration.

Additionally, the FFA solubility in PBS pH 6.8 at 37 ± 0.5°C was also measured in triplicate.

#### Cocrystal Solubility and Phase Solubility Diagrams (PSD) in DDW and PBS pH 4.5 in the Absence and Presence of a Predissolved Polymer or a Combination

For a 1:1 AB cocrystal without consideration of ionization of each component, its molar solubility $\left[{S}_{AB}\right]$ is calculated as [21]

1 $$\left[{S}_{AB}\right]=\sqrt{{K}_{sp}}=\sqrt{\left[{C}_{A}\right]\left[{C}_{B}\right]}$$

where ${K}_{sp}$ is the solubility product of the AB cocrystal, and $\left[{C}_{A}\right]$ and $\left[{C}_{B}\right]$ (μmol/mL) are the molar concentrations of A and B where the solution is in equilibrium with the solid residues including cocrystal solids. When the solution is in equilibrium with both the drug and cocrystal solids, it is called the eutectic point where the component concentrations in solution are designated as $\left[{C}_{A\_eut}\right]$ and $\left[{C}_{B\_eut}\right]$.

In order to determine the FFA-NIC cocrystal solubility, the physical mixtures of the FFA I and NIC solids and a constant 10 mL of the medium (i.e., DDW or PBS pH 4.5 in the absence and presence of a predissolved polymer, i.e., 0.2 mg/mL PVP-VA, 1 mg/mL PEG or 0.2 mg/mL SLP, or a combination, i.e., 0.2 mg/mL PVP-VA + 1 mg/mL PEG, 0.2 mg/mL PVP-VA + 0.2 mg/mL SLP), representing 10% w/w and 90% w/w of the total weight of a sample, were used. More than eight different ratios of the FFA I and NIC solids in the physical mixtures (Table S1 in the supporting materials) were prepared and then each of them was transferred into a 20 mL glass vial containing a magnetic stirrer (3 × 20 mm) with 10 mL of the solution prepared above. The samples were placed in the Polar Bear Plus Crystal (Cambridge Reactor Design Ltd, UK) and kept at 37 ± 0.5°C for 24 h. After that, the supernatant of each of the samples was separated from excess solids by centrifugation at 13,000 RPM for 1 min in an MSE Micro Centaur. HPLC was used to determine the concentrations of FFA and NIC. Solid residues obtained from the experiments were dried at room temperature and analysed by PXRD. If the FFA-NIC cocrystal solids were found in the solid residues of a sample ($i$), the molar concentrations of FFA ($\left[{C}_{FFA, i}^{exp}\right]$) and NIC ($\left[{C}_{NIC, i}^{exp}\right]$) (μmol/mL) obtained were used to determine the solubility product ${K}_{sp, i}^{exp}$ of FFA-NIC cocrystal by

2 $${K}_{sp, i}^{exp}= \left[{C}_{FFA, i}^{exp}\right]\times \left[{C}_{NIC,i}^{exp}\right]$$

The optimal solubility product ${K}_{sp }^{optimal}$ of FFA-NIC cocrystal is obtained by minimizing the sum square error (SSE) of the optimal value with each of the individual experimental values ${K}_{SP, i}^{exp}$.

3 $${K}_{sp }^{optimal}=min{\sum }_{i=1}^{n}{({K}_{sp }^{optimal}-{k}_{sp,i}^{exp})}^{2}$$

where $n$ is the total number of vials having the cocrystal solids in the residues.

In this work, the optimal value of ${K}_{sp }^{opitmal}$ was obtained using the inbuilt “fminbnd” function in MATLAB. If both the FFA-NIC cocrystal and FFA I solids were found in the solid residues of a sample, the measured concentrations of FFA ($\left[{C}_{FFA}^{exp}\right]$) and NIC ($\left[{C}_{NIC}^{exp}\right]$) were the transition concentrations at the eutectic point.

#### Particle Size Distribution

All samples (i.e., FFA, FFA-NIC cocrystal and FFA-TP cocrystal) used in the dissolution tests were slightly ground by a mortar and pestle and sieved by a 60-mesh sieve (below 250 μm) to reduce the influence of particle size on the dissolution rates. The particle size distributions of the test samples were measured using a SYNC Analyzer (MICROTRAC Retsch GmbH, Hann, Germany). The particle size distributions (D_10_, D_50_, D_90_ and mean particle size) of the solid particles were compared. The data were analysed by the Microtrac FLEX software (12.0.0.1).

#### Dissolution Tests of FFA, the Neat FFA-TP Cocrystal or FFA-NIC Cocrystal Powders in DDW in the Absence and Presence of a Predissolved Polymer or a Combination

*In vitro* dissolution tests were performed with a USP type II paddle apparatus (DT 126 light, Erweka GmbH, Germany) at 37 ± 0.5°C with a paddle speed of 50 rpm in 400 mL of DDW in the absence and presence of a predissolved polymer (i.e., 0.2 mg/mL of PVP-VA, 1 mg/mL of PEG, 0.2 mg/mL of SLP) or a combination of two polymers (i.e., 0.2 mg/mL PVP-VA + 1 mg/mL PEG, 0.2 mg/mL PVP-VA + 0.2 mg/mL SLP).

71.7 mg of FFA-NIC cocrystal powders with the equivalent of 50 mg FFA I powders were used in each of the dissolution tests. Samples of 1 ± 0.1 mL were withdrawn from the dissolution vessel at the predefined time points of 5, 10, 15, 30, 60, 120, 180 and 240 min. The supernatant of each of the samples was separated from the excess solids by centrifugation at 13,000 RPM for 1 min in an MSE Micro Centaur. HPLC was used to determine the concentrations of FFA and NIC in solution. Solid residues obtained from the experiments were dried at room temperature and analysed by PXRD. All experiments were repeated in triplicate.

For comparison, the above dissolution tests were also repeated using 50 mg of pure FFA I powders and 82.0 mg of FFA-TP cocrystal powders.

#### Dissolution Tests of the Powder Mixtures of the FFA-NIC Cocrystals with an Individual Polymer or a Combination in PBS pH 4.5 or PBS pH 6.8

Similarly, *in vitro* dissolution tests of the powder mixtures of 71.7 mg of the FFA-NIC cocrystals with an individual polymer or a combination in PBS pH 4.5 or PBS pH 6.8 were conducted in triplicate. The amount of a polymer used in the powder mixture was 80 mg of PVP-VA, 400 mg of PEG, or 80 mg of SLP, corresponding to their predissolved concentrations used in Section "Dissolution Tests of FFA, the Neat FFA-TP Cocrystal or FFA-NIC Cocrystal Powders in DDW in the Absence and Presence of a Predissolved Polymer or a Combination".

The solid residues obtained from the experiments in PBS pH 4.5 were dried at room temperature and analysed by PXRD.

#### Dissolution Performance Parameter (DPP)

DPP was used to evaluate the dissolution profile of the test powders in comparison to a reference system, which is defined as [5],

4 $$DPP=\frac{{AUC}_{C(t)}{-AUC}_{{C}_{R}(t)}}{{AUC}_{{C}_{R}(t)}}\times 100\mathrm{\%}$$

where ${AUC}_{C(t)}$ is the area under the curve (AUC) of a dissolution profile $C(t)$, indicating the amount of drug dissolved is maintained over the period of the dissolution time from 0 to* t*, and ${AUC}_{{C}_{R}(t)}$ is the AUC of a reference dissolution profile ${C}_{R}(t)$.

In comparison with the reference dissolution profile, a positive DPP value indicates an increased ability of the dissolved drug to be maintained in a dissolution medium, while a negative DPP value shows a lesser amount of the drug to be maintained in solution.

#### High Performance Liquid Chromatography (HPLC) Analysis

The sample concentration of FFA, NIC or TP in solution was determined by a Hewlett Packard series 1100 automatic HPLC system (Agilent Technologies, UK). A Roc C18 5 μm column, 150 × 4.6 mm (Restek, USA) was used at ambient temperature. FFA was detected by UV absorbance detection at a wavelength of 286 nm. The mobile phase used consisted of 15% water (including 0.5% formic acid) and 85% methanol, and the mobile phase flow rate was maintained at 0.5 mL/min. NIC and TP were detected by UV absorbance detection at a wavelength of 265 nm and 271 nm respectively, the mobile phase was composed of 15% water (including 0.5% formic acid) and 85% methanol, and the mobile phase flow rate was kept at 0.5 mL/min. The injection volume was 20 μL.

#### Powder X-ray Diffraction (PXRD)

The solids were scanned by D2 phaser diffractometer (Bruker U.K. Limited, Coventry, UK) with Cu-Kβ radiation operating at voltage of 30 kV and current of 10 mA. The scanning angle ranged from 2°-40° of 2θ. The scanning time per step was 0.4 s. The scan step size was 0.02° of 2θ.

#### Statistical Analysis

The differences in the FFA solubility in DDW and PBS pH 4.5 at 37°C in the absence and presence of a predissolved polymer or a combination were analysed by two-way Student's t-test with a significance level of 5%.

## Results

### Particle Size Distributions

Drug particle size, one of the important physicochemical properties of drugs, has a critical effect on drug release kinetics [22]. As a result, it is necessary to examine the particle size distributions of drug particles before dissolution tests. The particle size distributions of the representative FFA, FFA-NIC cocrystal and FFA-TP cocrystal powders used in the dissolution studies are shown in Table II. FFA, FFA-NIC cocrystal and FFA-TP cocrystal powders have similar D_10_ and D_50_ values. The D_90_ and mean values of FFA-NIC cocrystal distribution are the smallest, indicating a narrow size distribution (Figure S1 in the supporting materials). A possible reason is the needle-shaped morphology of the FFA-NIC cocrystals, leading to a smaller equivalent volume diameter, although all powders passed through the same size of the sieve aperture.

**Table II:** Particle Size Properties of Drug/Cocrystal Particles

Sieved particles	D10 (μm)	D50 (μm)	D90 (μm)	Mean (μm)
FFA < 250 μm	5.19	13.58	84.86	31.71
FFA-NIC < 250 μm	6.08	13.77	33.97	17.63
FFA-TP < 250 μm	5.25	12.32	56.64	23.92

Overall, the particle size distributions of the FFA and cocrystal powders of FFA-TP and FFA-NIC used in the study are comparable. Therefore, the difference in the dissolution performances among them was caused by the changes of the FFA molecule packings within their structures of crystalline lattices and interactions with the dissolution environments.

### Solubility

**Fig. 1:**
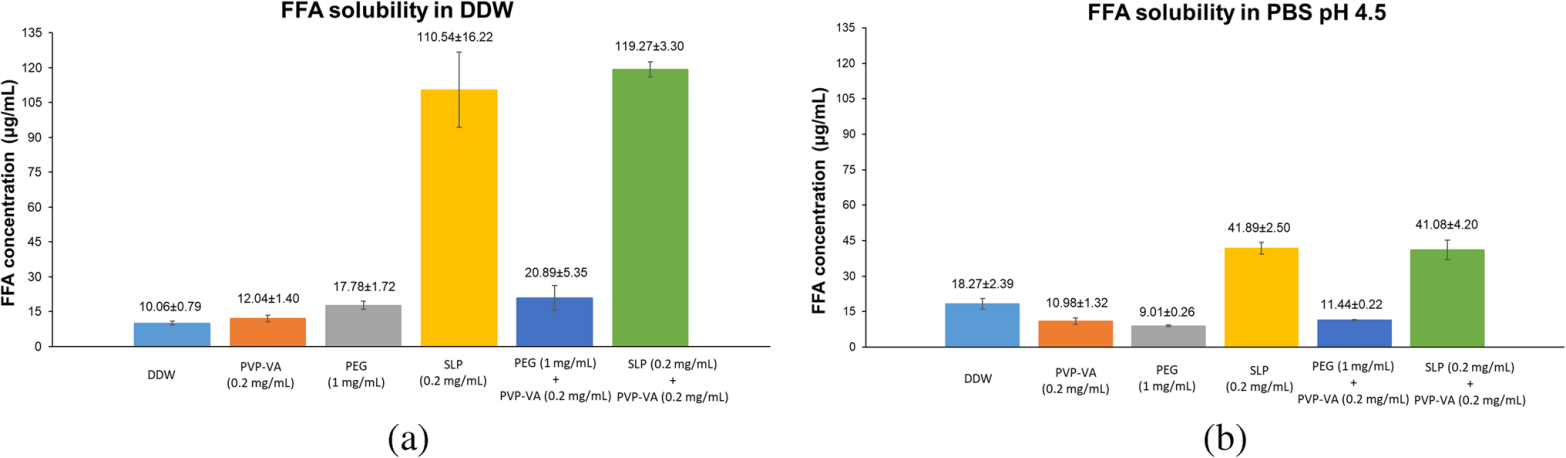
Solubility of FFA in: (**a**) DDW in the absence and presence of a predissolved polymer or a combination; (**b**) PBS pH 4.5 in the absence and presence of a predissolved polymer or a combination.

The FFA solubility in DDW and PBS pH 4.5 at 37°C in the absence and presence of a predissolved polymer or a combination are shown in Fig. 1. In Fig. 1(a), the FFA solubility in DDW at 37°C was 10.06 ± 0.79 μg/mL and remained the same in the presence of a predissolved polymer of 0.2 mg/mL PVP-VA (*P* > 0.05). In contrast, in the presence of a predissolved PEG or SLP in DDW, it shows different behaviour (P < 0.05). The FFA solubility was increased significantly to 110.54 ± 16.22 μg/mL with predissolved 0.2 mg/mL SLP whilst a small increase was observed in the presence of 1 mg/mL PEG, indicating that SLP is a stronger solubilizing agent. The FFA solubility in the presence of a combination of two predissolved polymers of 1 mg/mL PEG with 0.2 mg/mL PVP-VA also remained the same as that in the presence of PEG (*P* > 0.05), indicating there is no synergistic interaction between PEG and PVP-VA to solubilize the FFA molecules in DDW. A similar observation (*P* > 0.05) was applied to the combined predissolved polymers of 0.2 mg/mL SLP and 0.2 mg/mL PVP-VA when compared with that in 0.2 mg/mL SLP.

The FFA solubility in PBS pH 4.5 at 37°C was 18.27 ± 2.39 μg/mL in Fig. 1(b). Clearly, the FFA solubility decreased in the presence of a predissolved polymer of 0.2 mg/mL PVP-VA, or 1 mg/mL PEG while it increased significantly with predissolved 0.2 mg/mL SLP (P < 0.05). 0.2 mg/mL PVP-VA mixed with 1 mg/mL PEG in solution can slightly increase the FFA solubility when compared with the presence of a single polymer while the solubility remained the same (*P* > 0.05) in SLP predissolved PBS pH 4.5 in the absence and presence of PVP-VA. Overall, there is no synergistic interaction between PVP-VA with PEG or SLP to solubilize the FFA molecules in PBS pH 4.5.

It is worth noting that the PXRD results of the solid residues after the solubility tests (Figure S2 in the supporting materials) showed that the FFA solids remained the form I after the 24 h in various media, although nine polymorphic FFA solid forms were discovered [23]. Therefore, the value measured was the FFA I solubility.

As FFA is an acidic drug, its solubility will increase in a higher pH solution [24]. The equilibrium solubility of FFA in PBS pH 6.8 at 37°C was 741.15 ± 6.27 µg/mL.

Details of the solubility values can be found in Table S2 in the supporting materials.

### FFA-NIC Cocrystal Solubility and Phase Solubility Diagrams (PSDs)

Based on the PXRD results (Table S1 in the supporting materials), the optimal solubility product (${K}_{sp }^{optimal}$) of each group (Table III) was obtained based on Eq. (3) and the corresponding PSDs are plotted in Figure S3(a) (DDW) and Figure S3(b) (PBS pH 4.5) in the supporting materials. The FFA-NIC cocrystal solubility values were summarized in Table III, indicating that the cocrystal can significantly improve the parent drug FFA solubility (i.e., FFA neutral/free form). The impact of a polymer on the cocrystal solubility was different. PVP-VA in DDW or PBS pH 4.5 can significantly increase the FFA-NIC cocrystal solubility in comparison with PEG or SLP. A combination of PVP-VA and SLP can further enhance the FFA-NIC cocrystal solubility compared with the individual polymers. There is no significant change of the FFA-NIC cocrystal solubility in solution with PVP-VA or a combination of PVP-VA and PEG.

**Table III:** The Calculated Molar Solubility of FFA-NIC Cocrystal in Various Environment According to PSDs

solvents	DDW					PBS pH 4.5				
	${K}_{sp}$ (μmol^2^/mL^2^)	${S}_{AB}$ (μmol/mL)	Ratio of cocrystal solubility with parent drug	FFA concentration at eutectic point (μmol/mL)	Ratio of FFA concentration at eutectic point with parent drug	${K}_{sp}$ (μmol^2^/mL^2^)	${S}_{AB}$ (μmol/mL)	Ratio of cocrystal solubility with parent drug	FFA concentration at eutectic point (μmol/mL)	Ratio of FFA concentration at eutectic point with parent drug
Pure solution	94.76	9.73	2649.04	0.24	6.71	99.78	9.99	1535.91	0.16	2.46
with PVP-VA (0.2 mg/mL)	171.89	13.11	4805.23	0.83	23.20	132.58	11.51	2040.80	0.45	6.93
with PEG (1 mg/mL)	136.09	11.67	3218.49	0.16	4.47	104.01	10.20	1601.02	0.32	4.93
with SLP (0.2 mg/mL)	115.13	10.73	3218.49	0.16	4.47	115.13	10.73	1772.19	0.16	2.46
with PEG (1 mg/mL) + PVP-VA (0.2 mg/mL)	156.76	12.52	4382.27	0.26	7.27	141.26	11.89	2171.41	0.66	10.16
with SLP (0.2 mg/mL) + PVP-VA (0.2 mg/mL)	224.10	14.97	6264.77	1.11	31.03	158.72	12.60	2443.18	0.66	10.16

1) Ratio of the cocrystal solubility with the parent drug is defined as [S_AB_]/[S_A_] indicates the solubility advantage of the cocrystal

2) Ratio of FFA concentration at the eutectic point with the parent drug is define as $\left[{\mathrm{C}}_{\mathrm{FFA}\_\mathrm{eut}}\right]$/[S_A_] indicates FFA concentration improvement in solution.

3) [S_A_] is FFA solubility in DDW or PBS pH 4.5

The FFA concentrations at the eutectic points in different solutions were also shown in Table III. They are much higher than the FFA equilibrium solubility in DDW or PBS pH 4.5, but significantly less than the corresponding cocrystal solubility. The effect of a polymer or a combination on the FFA concentration at the eutectic point is similar to the cocrystal solubility.

### Dissolution Tests in DDW in the Absence and Presence of a Predissolved Polymer or a Combination

#### Dissolution Studies of FFA in DDW in the Absence and Presence of a Predissolved Polymer or a Combination (Fig. 2)

**Fig. 2:**
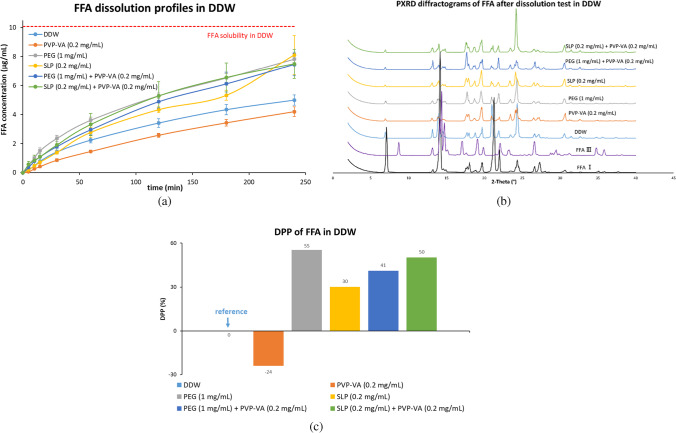
Dissolution performances of the FFA powders in DDW in the absence and presence of a predissolved polymer or a combination: (**a**) FFA powders dissolution profiles; (**b**) solid residues after tests; (**c**) DPP values.

It was found that the dissolution rate of the FFA powders in DDW was slow and the FFA concentration reached 4.99 μg/mL after 4 h, which was roughly half of its solubility [Fig. 2(a)]. In the presence of a predissolved polymer of PVP-VA, its dissolution performance was suppressed with the DPP value of -24% whilst in the presence of a predissolved polymer of PEG or SLP the FFA dissolution performance was slightly improved by 55% and 30% of the DPP values [Fig. 2(c)]. A combination of PVP-VA with PEG in DDW can reduce the dissolution performance of FFA-NIC cocrystal, in which the DDP value was reduced to 41% from 55% in the presence of PEG alone. Adding 0.2 mg/mL PVP-VA in the predissolved 0.2 mg/mL SLP solution, its DPP value increased to 50% from the original 30%. The overall FFA dissolution performance was poor and none of the FFA concentrations reached its solubility within 4 h.

The PXRD results of the solid residues collected after the FFA dissolution experiments were shown in Fig. 2(b), showing no phase transformation of FFA I during dissolution under different dissolution environments.

#### Dissolution Studies of FFA-TP Cocrystal in DDW in the Absence and Presence of a Predissolved Polymer or a Combination (Fig. 3)

**Fig. 3:**
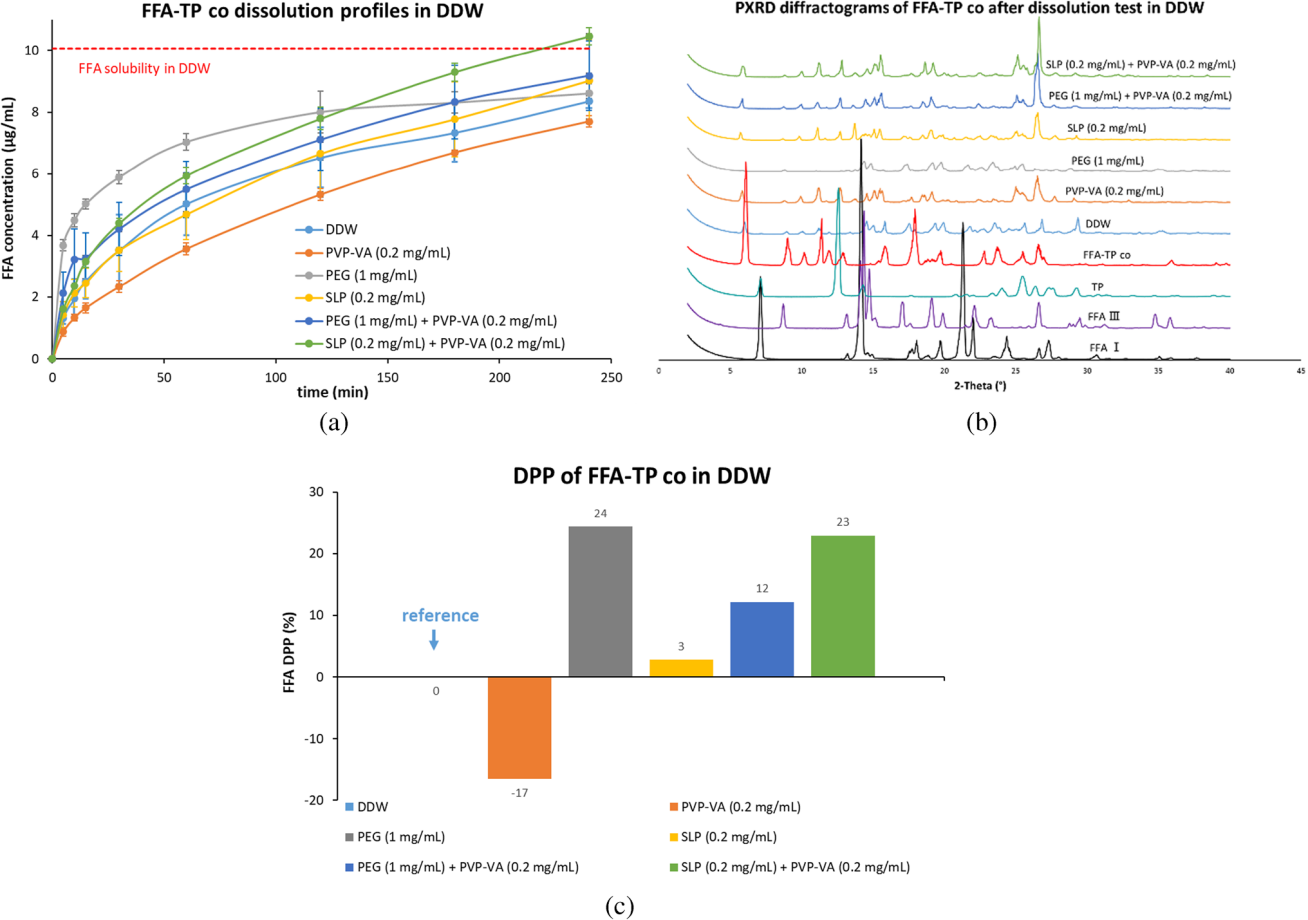
Dissolution performances of the FFA-TP cocrystal powders in DDW in the absence and presence of a predissolved polymer or a combination: (**a**) FFA concentration profiles; (**b**) Solid residues after tests; (**c**) DPP values.

Although the dissolution rate of FFA-TP cocrystal in DDW, as shown in Fig. 3(a), was increased in comparison with the parent drug of FFA I above (direct comparison is given in Figure S6 in the supporting materials), the FFA concentration in solution was still below its solubility after 4 h (i.e., 8.35 μg/mL). The presence of either PEG or SLP in DDW showed an increased FFA DPP value (i.e., 24% or 3%) whilst the dissolution rate of FFA from FFA-TP cocrystal in PVP-VA predissolved DDW was reduced, with the DPP value of -17% [Fig. 3(c)]. The FFA-TP cocrystal dissolution performance was depressed in a combination of PEG and PVP-VA in DDW in comparison with PEG alone, i.e., the DPP value was reduced to 12% from 24%. The DPP value of FFA-TP in the combination of SLP and PVP-VA predissolved DDW (23%) was slightly higher than a single individual polymer predissolved DDW.

The PXRD results [Fig. 3(b)] indicated that the solid residues after the dissolution tests were the same as the starting materials of FFA-TP cocrystal (Although there were minor differences, all of the characteristic peaks of FFA-TP cocrystal remained). The TP dissolution curve is shown in Figure S4(a) in the supporting materials.

#### Dissolution Studies of FFA-NIC Cocrystal in DDW in the Absence and Presence of a Predissolved Polymer or a Combination (Fig. 4)

**Fig. 4:**
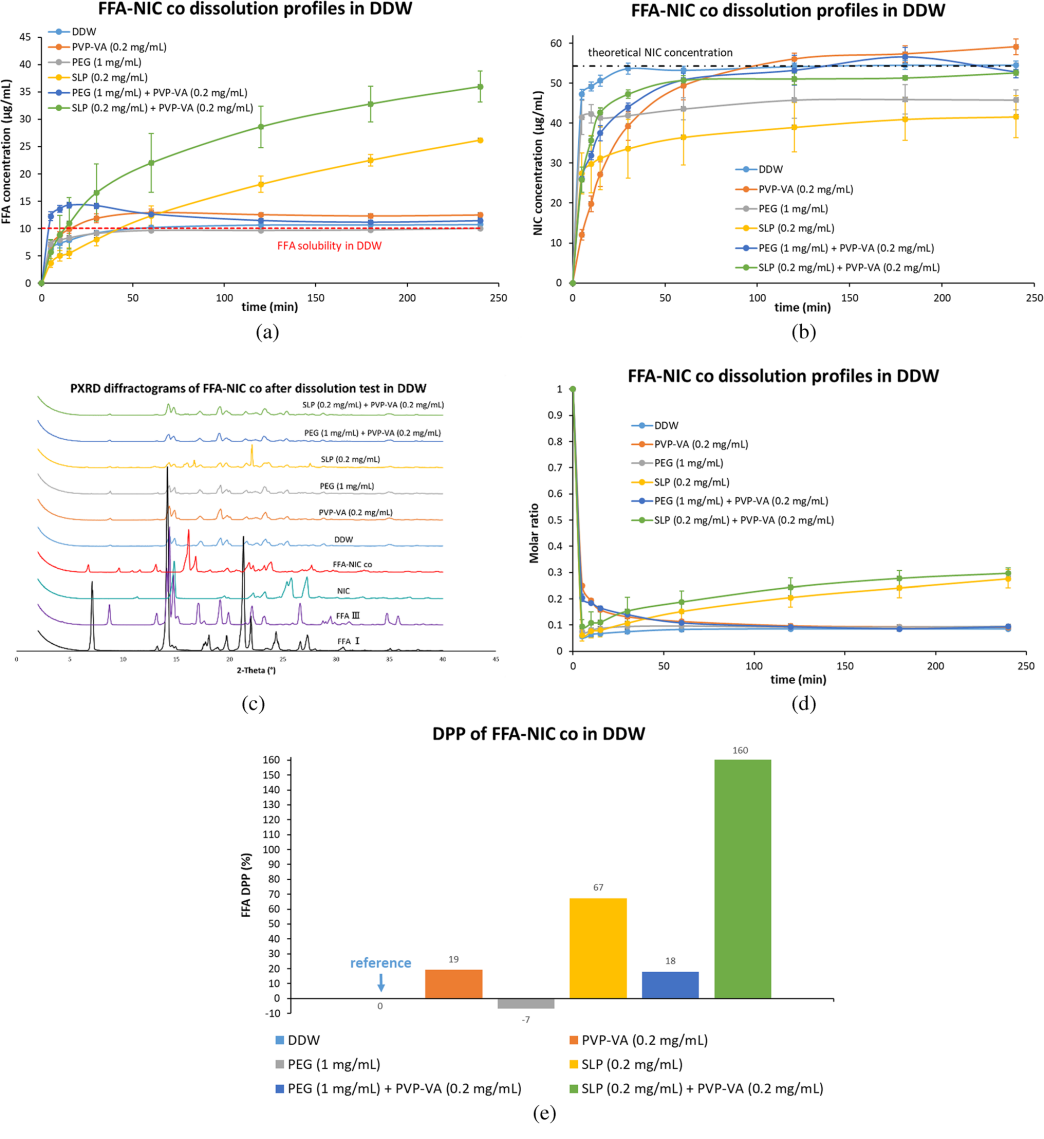
Dissolution performances of the FFA-NIC cocrystal powders in DDW in the absence and presence of a predissolved polymer or a combination: (**a**) FFA concentration profiles; (**b**) NIC concentration profiles; (**c**) solid residues after FFA-NIC cocrystal powder dissolution; (**d**) dynamic incongruent curves of molar concentrations of FFA vs. NIC in solution; (**e**) DPP values.

The FFA concentration quickly reached 10.23 μg/mL, which was slightly higher than its aqueous equilibrium solubility (i.e., 10.06 μg/mL) within 60 min and then stayed constantly at 10.78 μg/mL during the FFA-NIC cocrystal powder dissolution in DDW [Fig. 4(a)]. No supersaturated state of the FFA concentration was observed. There was no change of the dissolution profile of FFA-NIC cocrystal in DDW in the presence of predissolved 1 mg/mL PEG.

As shown in Fig. 4(a), the dissolution performances of FFA-NIC cocrystal were enhanced in the presence of predissolved 0.2 mg/mL PVP-VA with the DPP value of 19% or 0.2 mg/mL SLP with the DPP value of 67%. However, the effects of the polymers on the FFA release profiles were different. In 0.2 mg/mL PVP-VA predissovled DDW, the FFA dissolution rate was increased significantly at the beginning of the cocrystal dissolution and its solution concentration quickly reached its aqueous equilibrium solubility within 15 min. After that, the FFA concentration continued to increase to its maximum of 12.93 μg/mL at 60 min and then gradually decreased and stabilized at 120 min. In contrast, in the presence of SLP, the FFA release rate from FFA-NIC cocrystal was reduced considerably at the beginning of the cocrystal dissolution up to 30 min and then the FFA concentration in solution increased slowly, with a linear function of the dissolution time, to 26.18 μg/mL after 4 h.

A further enhancement of the dissolution performance of FFA-NIC cocrystal was observed in a combined PVP-VA with SLP predissolved DDW, i.e., the DPP value of 160% was observed in Fig. 4(e). Additionally, an increase in the FFA release rate was observed at the beginning of the dissolution. After 60 min, the FFA release rates in the presence and absence of PVP-VA in predissolved SLP solution are the same, showing two parallel curves in Fig. 4(a).

There was no obvious change of the DPP value under the dissolution conditions of the predissolved PVP-VA solution in the absence and presence of PEG in Fig. 4(e). However, the FFA dissolution profiles were different. In a combination of the predissolved PVP-VA and PEG, the supersaturated state of FFA concentration was rapidly generated within 5 min and it continued to increase at a slow rate to the maximum value of 14.28 µg/mL and subsequently decreased gradually to reach (11.44 μg/mL) and stabilize around its solubility at 240 min.

As shown in Fig. 4(b), NIC was released completely and reached its theoretical value (i.e., 54.28 μg/mL), in which the starting materials of the FFA-NIC cocrystals were dissolved completely, in PVP-VA or its combination with PEG or SLP predissolved DDW. In the presence of a single polymer of PEG or SLP, the release of NIC from the cocrystal powders was not complete, in which the NIC concentration was much lower than its theoretical value. In the meantime, the NIC release rates were also different under different environments. The quickest NIC release rate at the start of FFA-NIC cocrystal dissolution was observed in DDW in the absence or presence of PEG. The slowest NIC release rate was observed in PVP-VA predissolved DDW.

According to the PXRD results [Fig. 4(c)], the solid residues after the dissolution experiments in DDW with the absence or presence of predissolved polymers were FFA III, indicating that phase transform took place during the dissolution of the FFA-NIC cocrystal powders. Additionally, both FFA III and FFA-NIC cocrystal in the solid residues were presented after the FFA-NIC cocrystal dissolution in SLP predissolved DDW, supporting evidence that the NIC concentration was much lower than those in the other groups.

#### Dissolution Studies of FFA-NIC Cocrystal in DDW in the Presence of a Combination of Pre-Dissolved PVP-VA and SLP at Various Concentrations (Fig. 5)

**Fig. 5:**
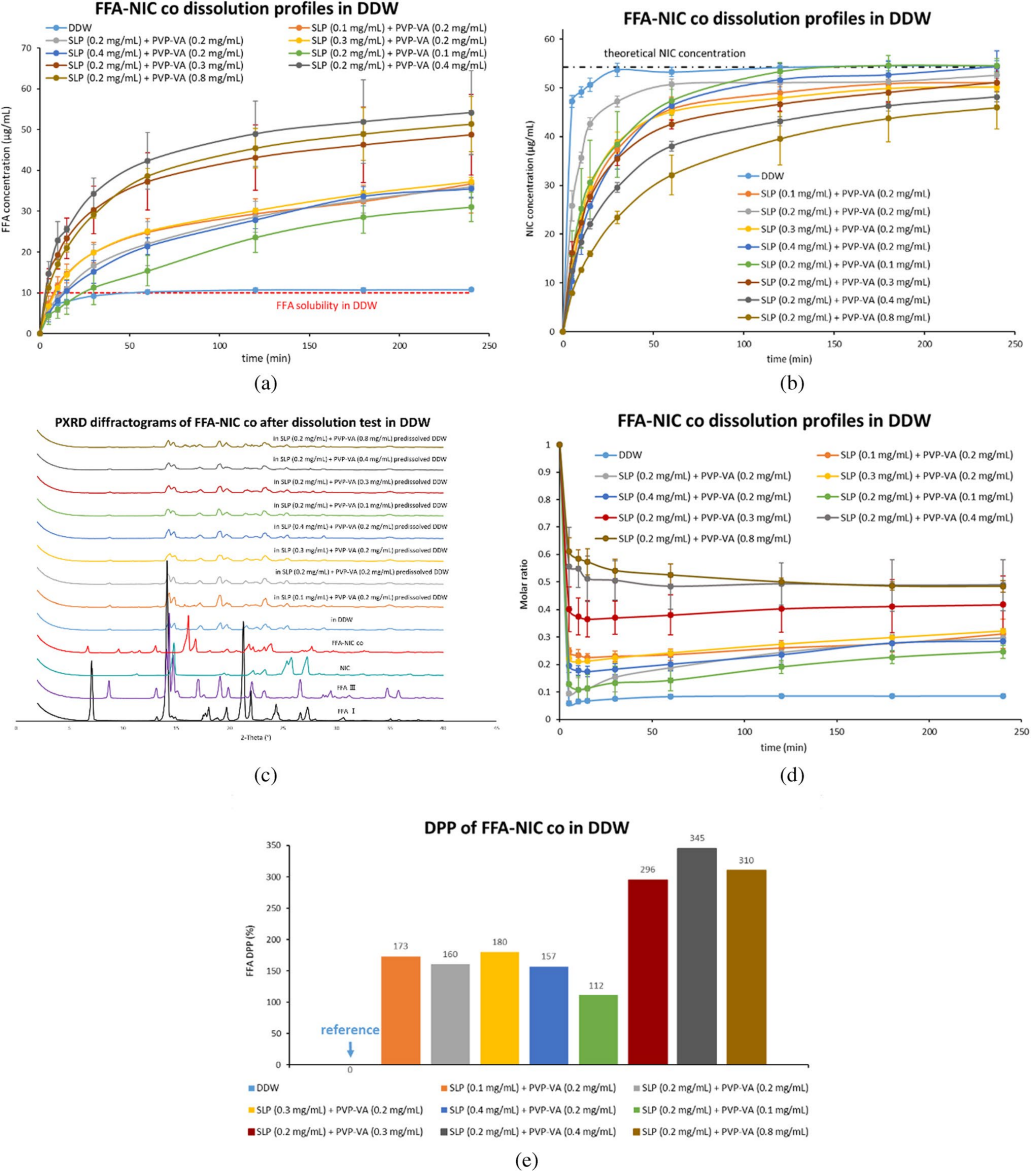
Dissolution performances of the FFA-NIC cocrystal powders in DDW in the presence of a combination of predissolved PVP-VA and SLP at various concentrations: (**a**) FFA concentration profiles; (**b**) NIC concentration profiles; (**c**) solid residues after tests; (**d**) dynamic incongruent curves of molar concentrations of FFA vs. NIC in solution; (**e**) DPP values.

At a fixed concentration of 0.2 mg/mL PVP-VA predissolved in DDW, the FFA release rate from FFA-NIC cocrystal was not affected significantly by varying the predissolved SLP concentration (i.e., 0.1, 0.2, 0.3 and 0.4 mg/mL) [Fig. 5(a)]. In contrast, varying the predissolved PVP-VA concentration (i.e., 0.1, 0.2, 0.3, 0.4 and 0.8 mg/mL) at a fixed predissolved SLP concentration of 0.2 mg/mL resulted in different cocrystal dissolution behaviours. It was observed that increasing PVP-VA concentration from 0.1 up to 0.4 mg/mL in solution led to an increased FFA dissolution rate at the beginning of the cocrystal dissolution and a further increase in PVP-VA concentration to 0.8 mg/mL resulted in a decrease in the dissolution performance [Fig. 5(a)]. These findings agree well with the DPP values [Fig. 5(e)] which were in the order of 0.2 mg/mL SLP + 0.1 mg/mL PVP-VA (112%) < 0.2 mg/mL SLP + 0.2 mg/mL PVP-VA (160%) < 0.2 mg/mL SLP + 0.3 mg/mL PVP-VA (296%) < 0.2 mg/mL SLP + 0.8 mg/mL PVP-VA (310%) < 0.2 mg/mL SLP + 0.4 mg/mL PVP-VA (345%).

The PXRD results [Fig. 5(c)] illustrated that the solid residues collected after the dissolution tests were FFA III alone except for the test in a combination of SLP (0.2 mg/mL) and PVP-VA (0.8 mg/mL) predissolved DDW where the mixtures of FFA III and FFA-NIC cocrystal were obtained. The result is consistent with the incomplete release of NIC [Fig. 5(b)] in this group.

### Dissolution Tests of Powder Mixtures of FFA-NIC Cocrystal with Individual Single Polymers or a Combination

#### Non-Sink Conditions Dissolution Tests in PBS pH 4.5 (Fig. 6)

**Fig. 6:**
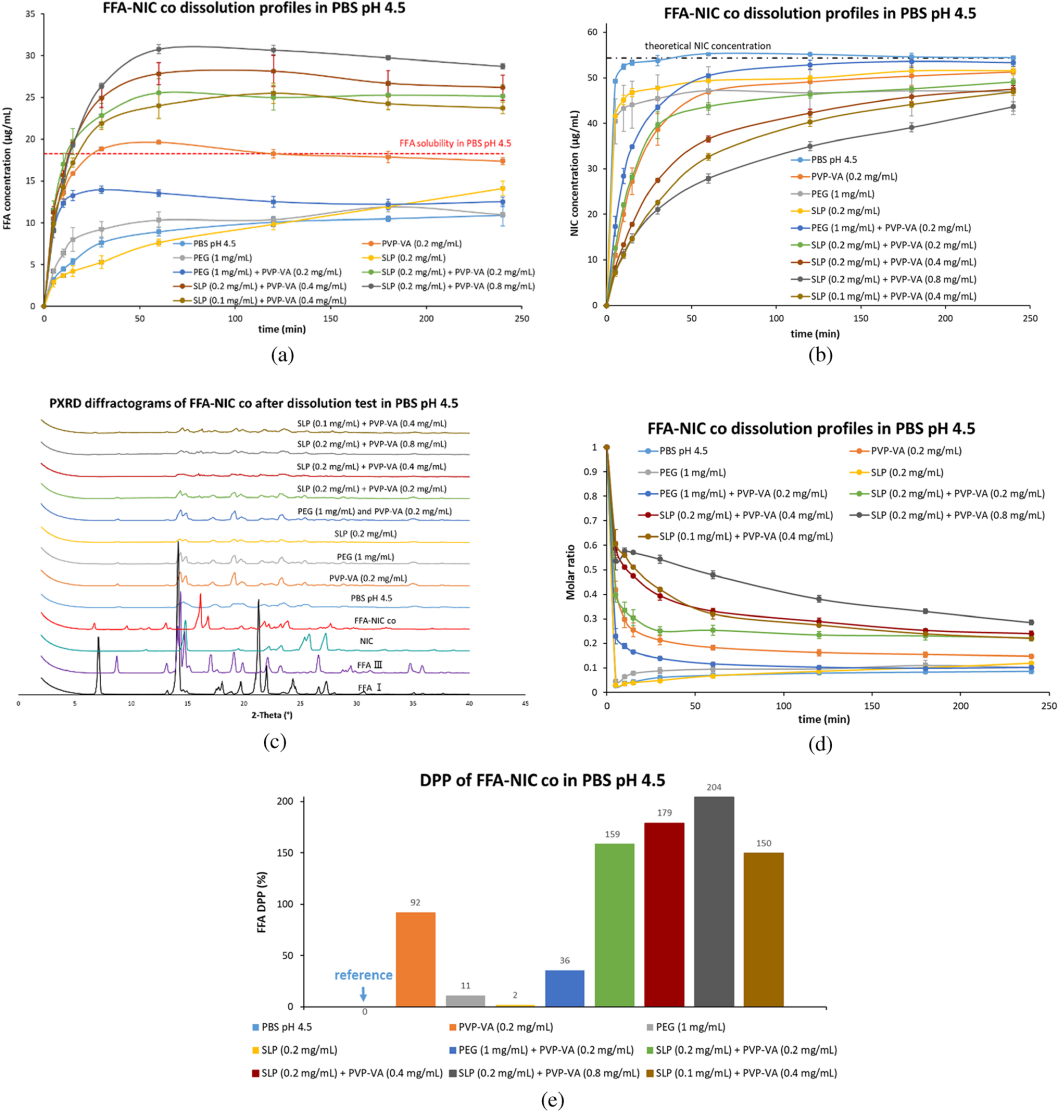
Dissolution performances of the physical mixtures of the FFA-NIC cocrystal powders with individual single polymers or a combination in PBS pH 4.5: (**a**) FFA concentration profiles; (**b**) NIC concentration profiles; (**c**) solid residues after tests; (**d**) dynamic incongruent curves of molar concentrations of FFA vs. NIC in solution; (**e**) DPP values.

In order to compare the dissolution behaviours of FFA-NIC cocrystal in different environments under non-sink conditions, PBS pH 4.5 was selected as the dissolution medium due to the low FFA solubility [i.e., 18.27 ± 2.39 μg/mL in Fig. 1(b)]. Flufenamic acid is an ampholyte, having a basic group with pKa1 of 2.92 ± 0.01, an acidic group with pKa2 of 4.84 ± 0.04 and isoelectric point pH of 3.88 ± 0.03 [24]. At pH 4.5, FFA is largely in the neutral form (i.e., more than 75%). Therefore, for simplicity, the FFA-NIC cocrystal solubility was calculated by Eq. (1) without considering the ionization of each component. The FFA release profiles from the FFA-NIC cocrystals mixed with a single polymer or a combination in PBS pH 4.5 are shown in Fig. 6(a). There was no significant improvement of the FFA DPP value for the mixtures of the FFA-NIC cocrystal with PEG (DPP value of 11%) or SLP (DPP value of 2%) in Fig. 6(e). In contrast, an enhanced dissolution performance was obtained with the presence of PVP-VA (DPP value of 92%) in the powder mixtures, where a maximum supersaturated FFA concentration of 19.65 μg/mL was reached at 60 min and declined gradually to 17.38 μg/mL, which was near to its solubility, at the end of the test.

A combination of PVP-VA and SLP with the FFA-NIC cocrystals showed a further enhanced dissolution performance in PBS pH 4.5 [Fig. 6(a)]. Similar to a combination of predissolved PVP-VA and SLP in DDW, varying PVP-VA in the powder mixtures led to a significant change in the FFA-NIC cocrystal dissolution performance. The best dissolution performance was achieved with the highest concentration of 0.8 mg/mL PVP-VA in the mixtures. According to the FFA DPP values of the dissolution of FFA-NIC cocrystal in PBS pH 4.5 [Fig. 6(e)], the values were in the order of 0.2 mg/mL SLP (2%) < 0.2 mg/mL PVP-VA (92%) < 0.4 mg/mL PVP-VA + 0.1 mg/mL SLP (150%) < 0.2 mg/mL PVP-VA + 0.2 mg/mL SLP (159%) < 0.4 mg/mL PVP-VA + 0.2 mg/mL SLP (179%) < 0.8 mg/mL PVP-VA + 0.2 mg/mL SLP (204%).

Surprisingly, FFA-NIC cocrystal mixed with the combination of PEG and PVP-VA led to a reduced dissolution performance (36%) in comparison with PVP-VA alone (92%).

The PXRD results [Fig. 6(c)] indicated that FFA III solids were crystallised from the solution from all of the dissolution tests in PBS pH 4.5. Additionally, the FFA-NIC cocrystals were also found in the solid residues of the groups of SLP (0.1 mg/mL) + PVP-VA (0.4 mg/mL) and SLP (0.2 mg/mL) + PVP-VA (0.8 mg/mL) and the corresponding NIC release rates were slower, shown in Fig. 6(b).

#### Sink Conditions Dissolution Tests in PBS pH 6.8 (Fig. 7)

**Fig. 7:**
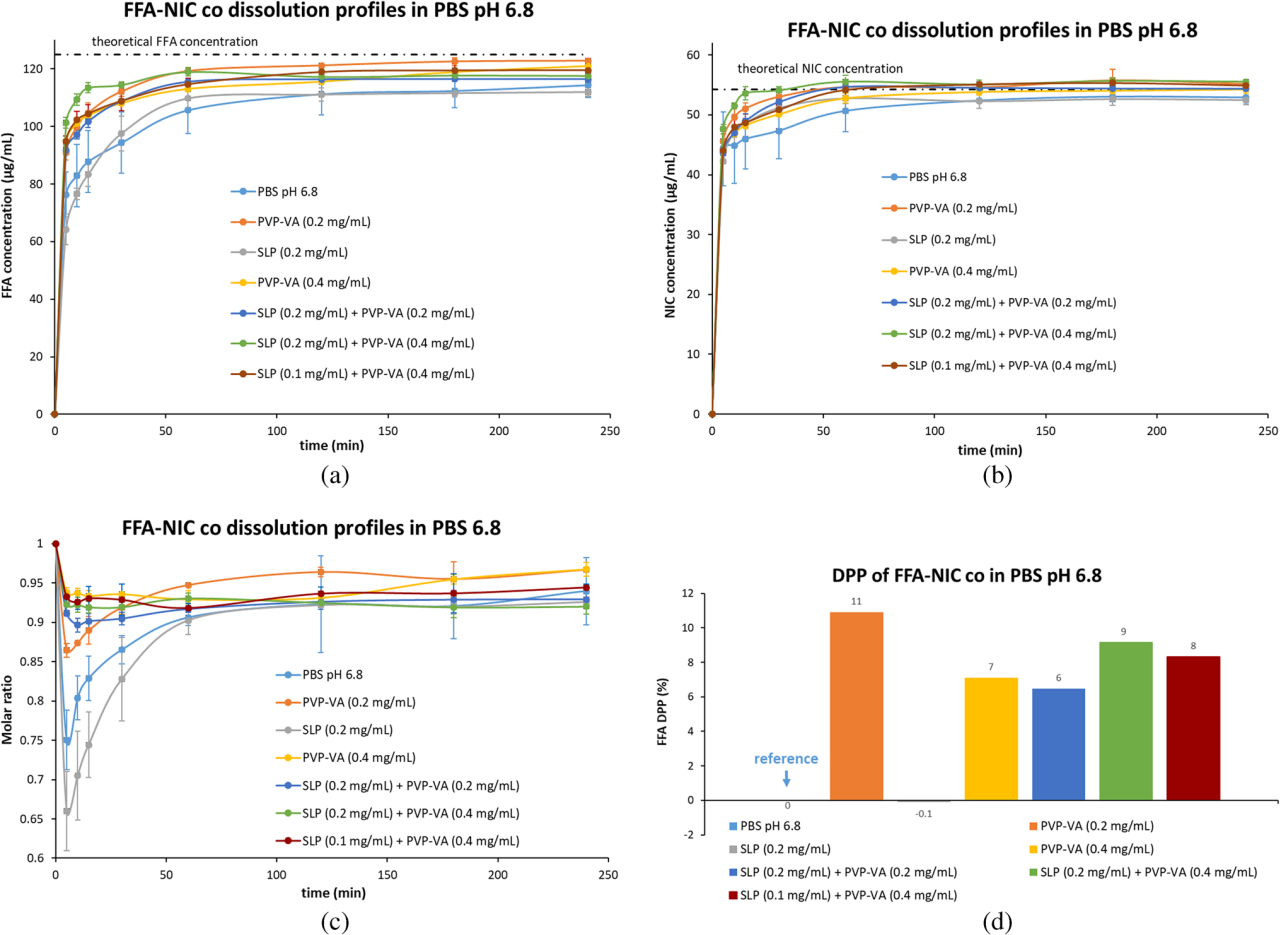
Dissolution performances of the physical mixtures of the FFA-NIC cocrystal powders with individual single polymers or a combination in PBS pH 6.8: (**a**) FFA concentration profiles; (**b**) NIC concentration profiles; (**c**) dynamic incongruent curves of molar concentrations of FFA vs. NIC in solution; (**d**) DPP values.

As the equilibrium solubility of FFA in PBS pH 6.8 at 37°C was 741.15 ± 6.27 μg/mL, the 400 mL dissolution medium used in the test can dissolve around 300 mg of FFA. Thus, the dissolution tests of the FFA-NIC cocrystal powders with an equivalent of 50 mg FFA used in PBS pH 6.8 at 37°C were conducted under sink conditions. The dissolution tests of the FFA-NIC cocrystal powders mixed with PVP-VA, SLP or their combination were examined. The physical mixtures of FFA-NIC cocrystal with SLP cannot improve its dissolution performance, shown in Figs. 7(a) & (d). Mixing FFA-NIC cocrystal with PVP-VA or a combination of PVP-VA and SLP at different concentrations displayed a comparable improved dissolution performance in comparison with the neat cocrystal powders in PBS pH 6.8 and their FFA DPP values are shown in Fig. 7(d) in the order of 0.2 mg/mL SLP (-0.1%) < 0.2 mg/mL PVP-VA + 0.2 mg/mL SLP (6%) < 0.4 mg/mL PVP-VA (7%) < 0.4 mg/mL PVP-VA + 0.1 mg/mL SLP (8%) < 0.4 mg/mL PVP-VA + 0.2 mg/mL SLP (9%) < 0.2 mg/mL PVP-VA (11%). The coformer NIC release rate shows the same trend as its corresponding FFA release rate under different conditions, shown in Fig. 7(b).

Due to the high solubility of both FFA and NIC in PBS pH 6.8 at 37°C, no solid residue can be collected after the dissolution after 4 h.

## Discussion

Cocrystallisation of a poorly water-soluble API with soluble coformers has been extensively exploited as a strategy for improving its dissolution performance over the last two decades [25]. As a cocrystal form of the poorly water-soluble API has higher free energy compared to the pure crystalline API counterpart, a transient but high supersaturated solution concentration (i.e., kinetic solubility), which is significantly greater than the equilibrium solubility of the pure parent drug, can be generated. Thus, improved drug absorption and increased bioavailability can be achieved. At the same time, the API supersaturation leads to higher instability of the API in solution as the thermodynamic driving force always favours a transformation towards a lower energy crystalline state, negating the solubility advantage [26]. It is critical to design an enabling cocrystal formulation through selection of effective excipients as precipitation inhibitors to maintain the elevated drug supersaturation [27]. However, due to lack of a mechanistic understanding of cocrystal dissolution, the cocrystal formulation development is largely empirical.

### Cocrystal Solubility vs. Maximum API Concentration in Solution

It has been frequently mentioned that the advantage of pharmaceutical cocrystals is to increase the solubility of poorly water-soluble drug compounds. A definition of pharmaceutical cocrystal solubility without consideration of ionization of each component is given in Eq. (1), which is based on the solubility product of the concentrations of the API and coformer in solution. In some cases, more than a 1000-fold increase of the FFA solubility was achieved by FFA-NIC cocrystal (Table III), which is unrealistic. As the therapeutic improvement of an API is determined by its maximum concentration in solution alone, a definition of the maximum therapeutic concentration of API in a cocrystal system would be more appropriate to evaluate its solubility advantage.

**Fig. 8:**
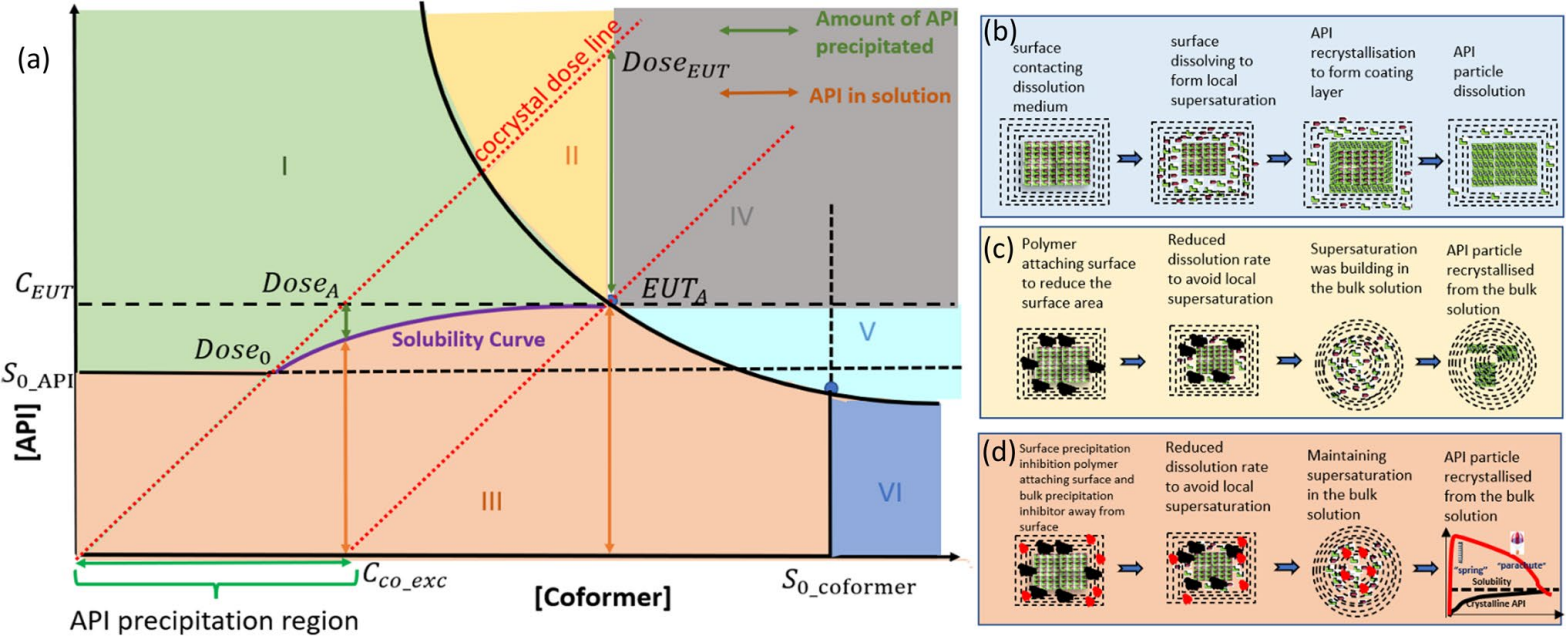
Dissolution mechanism of a cocrystal with surface precipitation of the parent drug: (**a**) dose effects (Zone I: the solution is supersaturated with drug; Zone II: the solution is supersaturated with both API and cocrystal; Zone III: Homogeneous liquid phase containing API and coformer; Zone IV: the solution is supersaturated with cocrystal at eutetic point and the rest is cocrystal; Zone V: the solution is superdaturated with coformer and cocrystal; Zone VI: solution is supersaturated with coformer); (**b**) neat cocrystal dissolution and parent drug precipitation; (**c**) cocrystal dissolution with surface precipitation inhibitor and parent drug recrystallisation in bulk solution; (**d**) cocrystal dissolution with a combination of surface and bulk precipitation inhibitors.

A cocrystal PSD in Fig. 8(a) illustrates not only the different solid regions but also the equilibrium composition of the solution, in which the FFA concentration (${C}_{FFA\_eut}$) at the eutectic point A (${EUT}_{A}$) is the maximum concentration which can be obtained in the cocrystal solution system [21, 28, 29]. Therefore, potentially a 30-fold (in DDW) or tenfold (in PBS pH 4.5) increase in the FFA solubility could be achieved by the FFA-NIC cocrystal system based on the API therapeutic solubility. According to the PSD, the drug concentration in solution is a function of the cocrystal dose used. For a neat cocrystal system in Fig. 8(a), to achieve the maximum API concentration (${C}_{EUT}$), it requires a minimum dose of ${Dose}_{EUT}$, which could be extremely high for soluble cocrystals, such as FFA-NIC cocrystal in Tables S1 in the supporting materials. In the meantime, a large amount of the parent drug will be precipitated from the solution, illustrated in Fig. 8(a). A potential solution to achieve the maximum API concentration (${C}_{EUT}$) with a minimum dose (${Dose}_{A}$) is to present excess coformer (${C}_{co\_exc}$) in solution to eliminate precipitation through a depression of the cocrystal dissolution [30, 31]. This also requires a high amount of the excess coformer predissolved in solution. Hence, none of the approaches is practical for developing an effective cocrystal formulation.

Without excess coformer in solution, any dose, which is greater than ${Dose}_{0}$, will lead to precipitation of the parent drug shown in Fig. 8(a), and then incomplete absorption. Thus, a key obstacle for a cocrystal formulation development is to prevent the API recrystallization from solution without excess coformer, which is complex.

### Cocrystal Surface Precipitation Mechanism

In our previous studies, we have demonstrated that the API precipitation occurred once the surface of a cocrystal contacts the dissolution medium [5, 6, 32]. Clearly, the surface precipitation of the parent API is induced by the local supersaturation (or microenvironment) around the dissolving cocrystal particle surface (e.g. FFA-NIC cocrystal). This process happens rapidly due to API heterogeneous nucleation promoted by the dissolving surface as a foreign particle. Consequently, an outer layer of the crystalline API is coated on the dissolving cocrystal particle [5]. Therefore, the dissolution of a cocrystal with surface precipitation of the parent drug can be described by four consecutive steps in Fig. 8(b) as: i) the cocrystal surface contacting the dissolution medium; ii) the cocrystal surface dissolving; iii) the parent drug precipitation on the dissolving surface; and iv) the parent drug particles redissolving. The cocrystal dissolving and parent drug precipitation were supported by the PXRD results in Figs. 4(c) & 6(c), indicating that FFA III was recrystallised from solution as it is stable below 42°C whereas FFA I is the stable form above 42°C [33]. Due to the surface precipitation of the parent drug FFA III on the dissolving FFA-NIC cocrystal particles, the dissolution curve obtained was actually from the precipitated FFA III solids. It was unsurprising that no “spring” supersaturation was observed in the kinetic solubility curve of FFA under non-sink dissolution conditions of the FFA-NIC cocrystal dissolution (i.e., DDW and PBS pH 4.5) where the FFA concentration was stabilised once reaching its equilibrium solubility [Figs. 4(a) & 6(a)]. FFA recrystallisation during the FFA-NIC cocrystal dissolution can also been indicated by an incongruent indicator $k(t)$ which is the ratio of the molar concentrations $\left[C\right]$ of FFA and NIC in solution, defined as

5 $$k(t)=\frac{\left[{C}_{API}(t)\right]}{\left[{C}_{Coformer}(t)\right]}=\frac{{C}_{API}(t)/{m}_{API}}{{C}_{Coformer}(t)/{m}_{Coformer}}$$

where ${C}_{(t)}$ is the concentration at the time $t$ and $m$ is the molecular weight. Since the crystal unit cell as the smallest building block is detached from the dissolving surface, in which all molecules enter into the solution simultaneously during dissolution [34], it is expected that the value of the incongruent indicator $k(t)$ is close to 1. In fact, in DDW or PBS pH 4.5 under non-sink conditions shown in Figs. 4(d) & 6(d), its value was quickly dropped to 0.06 or 0.03 at 5 min and then slowly increased to 0.08 or 0.07 at 60 min during the course of the cocrystal dissolution, indicating that the FFA recrystallisation first took place and subsequently started to redissolve.

As the surface precipitation of the parent API is induced by the local supersaturation, recrystallisation of API is expected to occur during a cocrystal dissolution under sink conditions as well, in which the cocrystal dose is less than ${Dose}_{0}$ shown in Fig. 8(a). Under the sink conditions of PBS pH 6.8, the FFA release rate between 5 to 10 min was reduced significantly in comparison with the initial rate in Fig. 7(a), indicating the transformation of the dissolving solid particles. It was further confirmed by the evolution of the incongruent indictor $k(t)$ which was reduced to 0.75 at 5 min, indicating the solid form precipitation from solution, and then gradually increased to 0.94 at the end of dissolution, indicating all of the recrystallised solids have been redissolved in solution [Fig. 7(c)].

### Surface Precipitation Inhibition by a Single Polymer

The predissolved PVP-VA in solution can be adsorbed on the dissolving cocrystal particles to reduce the cocrystal surface area directly contacting the dissolution medium, leading to a reduced cocrystal dissolution rate and the local drug supersaturation [6]. Hence, PVP-VA was a good surface precipitation inhibitor to prevent the FFA precipitation on the dissolving surface of FFA-NIC cocrystal. Here, we have further demonstrated that surface precipitation inhibition can be achieved when PVP-VA was physically mixed with the FFA-NIC cocrystal powders. In the predissolved or physically mixed 0.2 mg/mL PVP-VA in DDW or PBS pH 4.5 under non-sink conditions, a typical “spring” and “parachute” were observed [Fig. 4(a) & Fig. 6(a)]. From the practical point of view, the implications of these results are significant, showing physical mixtures of a small amount of PVP-VA powders in a FFA-NIC cocrystal formulation can reveal the cocrystal dissolution advantage for absorption and bioavailability improvement [i.e., Fig. 4(e) & Fig. 6(e)]. Under sink conditions of PBS pH 6.8, the FFA release performance from the mixtures was also improved with the DPP value of 11% by preventing the surface precipitation, showing a small reduction of the incongruent constant $k(t)$ as 0.75 at 5 min and then quickly increased above to 0.94 at 60 min.

PEG or SLP alone cannot reduce or prevent the surface precipitation of FFA during FFA-NIC cocrystal dissolution [Fig. 4(a) & 6(a)], showing a significant drop of the incongruent indicator at the beginning of the dissolution in the polymer predissolved DDW [Fig. 4(d)] or physically mixed powders in PBS pH 4.5 [Fig. 6(d)]. Actually, SLP enhanced the surface precipitation, showing a significant reduction of the FFA release rate in DDW or PBS pH 4.5 compared with that in pure solution or with PEG [Figs. 4(a) & 6(a)]. This has been further supported by the incongruent indicators [Figs. 4(d) & 6(d)], showing the lowest value at the beginning of dissolution compared to the other conditions. Therefore, a typical “spring and parachute” kinetic solubility profile of FFA was not observed. Additionally, as SLP increased the FFA solubility in DDW significantly shown in Fig. 1(a), the original “non-sink conditions” of the FFA-NIC dissolution test have been changed even though it has not met the requirement of “sink conditions”. As a result, it was observed that the FFA concentration in solution was increasing gradually due to redissolving of the recrystallised FFA III solids during the course of the test [Fig. 5(a)].

### Dissolution Performance Enhancement Through Optimising a Combination of Polymers for Synergistic Precipitation Inhibition

In the above experiments, PVP-VA played an important role to avoid surface precipitation for an enhanced FFA DPP value from the dissolution of FFA-NIC cocrystal. However, once the built-up FFA concentration in the bulk solution was higher than its equilibrium solubility, the bulk recrystallisation took place because at this point the FFA molecules cannot be sustained in solution by the coformer of NIC due to its low concentration shown in Fig. 8(c). Thus, a single polymer could not fulfil all requirements, for example, a higher AUC or a “spring” effect [Fig. 8(c)]. Actually, PVP-VA was not an effective bulk solution precipitation inhibitor [15]. A combination of two polymers could provide a useful solution to optimise the effectiveness of a cocrystal formulation to maximise its dissolution performance, in particular by reducing the amount of polymer used in the formulation. A higher percentage of polymer in the formulation can result in large tablets or even multiple doses, which is inconvenient to patients or even poor compliance.

In this study, PVP-VA was used as a precipitation inhibitor to prevent the surface precipitation of FFA whilst the other polymer PEG or SLP functioned as an effective bulk solution precipitation inhibitor to maintain the FFA molecules in solution shown in Fig. 8(d). Obviously, a combination of PVP-VA (0.2 mg/mL) + PEG (1 mg/mL) resulted in an unfavourable synergistic effect. In DDW with a combination of the predissolved two polymers, the “spring” effect was improved while the “parachute” effect was reduced, shown in Fig. 4(a). Thus, the overall DPP value was almost the same i.e.,19% for PVP-VA and 18% for PVP-VA + PEG [Fig. 4(e)]. In PBS pH 4.5, both the “spring” and “parachute” effects [Fig. 6(a)] were reduced for the dissolution of the FFA-NIC cocrystals mixed with the two polymers, resulting in a reduced DPP value of 36% from 92% mixed with PVP-VA alone [Fig. 6(e)].

In contrast, a combination of PVP-VA and SLP resulted in a synergistic effect. In DDW with a combination of the predissolved PVP-VA (0.2 mg/mL) and SLP (0.2 mg/mL), a 160% increase in DPP was observed compared with the individual polymers, i.e., 19% for PVP-VA and 67% for SLP [Fig. 4(e)]. Additionally, the synergistic effect depended on the individual polymer concentrations as well [Fig. 5(a)]. At a fixed PVP-VA concentration of 0.2 mg/mL, there was no significant change in the DPP value by varying SLP concentration from 0.1 mg/mL to 0.4 mg/mL [Fig. 5(e)]. The reason behind this was a stronger solubilizing ability of SLP for the FFA molecules in solution (solubility curve in Figure S5 in the supporting materials), in which all of the released FFA molecules can be sustained in solution at the concentrations of SLP selected in the study. Therefore, there was no “parachute” behaviour in the dissolution profiles in Fig. 5(a). The overall dissolution performance of FFA-NIC cocrystal was dominated by the concentration of PVP-VA used. At a fixed SLP concentration of 0.2 mg/mL, the FFA DPP value was increased with increasing the PVP-VA concentration from 0.1 mg/mL to 0.4 mg/mL, indicating an increased FFA release rate due to surface precipitation inhibition. A further increase of PVP-VA concentration to 0.8 mg/mL led to a reduced DPP value, indicating that an optimal PVP-VA concentration in the combination was required to maximise the synergistic effect. A similar trend of the FFA dissolution profiles from the physical mixtures of FFA-NIC cocrystal with both PVP-VA and SLP was observed in PBS pH 4.5 [Figs. 6(a) & (e)]. As the non-sink dissolution conditions were not changed at the 0.2 mg/mL SLP used in the study, the FFA dissolution profile followed the typical “spring and parachute” behaviour. The highest FFA DPP value of 204% was achieved when the FFA-NIC cocrystals were mixed with 0.8 mg/mL PVP-VA and 0.2 mg/mL SLP. For the FFA-NIC cocrystal dissolution under sink conditions in PBS pH 6.8, it was not required to prevent the bulk FFA precipitation so that no significant change of the FFA DPP value was observed when mixing PVP-VA or a combination of PVP-VA + SLP shown in Fig. 7(e). As no synergistic effect of a combination of PVP-VA and SLP on the equilibrium solubility FFA (Fig. 1), it was expected that the change of the dynamic FFA solubility curve from the cocrystal was caused by the individual polymer inhibition effects.

Finally, it was worth noting that the dissolution behaviour of a cocrystal with bulk precipitation of the parent drug, (FFA-TP) was similar to its parent drug of FFA I in Figure S6 in the supporting materials. The surface precipitation inhibitor should not be included in the formulation as it reduced the cocrystal dissolution rate [Fig. 3(a)]. Furthermore, it should not be expected that a typical “spring” and “parachute” of the parent drug from a cocrystal with bulk precipitation of the parent drug is observed even if a bulk precipitation inhibitor is included in the formulation.

## Conclusion

In this study, the dissolution performance of a highly soluble FFA-NIC cocrystal has been systematically studied under both non-sink and sink dissolution environments with predissolved or powder mixed a single polymer (i.e., PVP-VA, PEG, SLP) or binary polymers combinations (i.e., PVP-VA + PEG, and PVP-VA + SLP). The impact of the polymers on the drug release rates from the FFA-NIC cocrystal formulations has been examined. To quantitatively evaluate the performance of different cocrystal formulations, the FFA DPP value based on the AUC of the *in vitro* kinetic solubility profile has been used to correlate the corresponding potential bioavailability enhancement. The insights into the cocrystal dissolution mechanisms have been revealed. It is shown that the dissolution of a cocrystal with surface precipitation of the parent drug can be described by four consecutive steps as: i) the cocrystal surface contacting the dissolution medium; ii) the cocrystal surface dissolving; iii) the parent drug precipitation on the dissolving surface; and iv) the parent drug particles redissolving. A single polymer of PVP-VA as a surface precipitation inhibitor prevented the FFA surface precipitation for an enhanced dissolution performance of FFA-NIC cocrystal. Unfortunately, it cannot sustain the supersaturated FFA concentration in the bulk solution. Thus, a single polymer of PVP-VA could not fulfil all requirements, for example, a higher AUC or a “spring” effect. A combination of two polymers of PVP-VA and SLP has shown a synergistic inhibition effect to maximise the dissolution advantage of the surface cocrystal of FFA-NIC, in particular by reducing the amount of polymer used in the formulation. Our results also highlight that the type of polymers and their concentration combinations either in pre-dissolved solution or in physical mixtures need to be optimised to maximise the kinetic solubility curve of cocrystal in solution.

## Supplementary Information

Below is the link to the electronic supplementary material.

Supplementary file1 (DOCX 2797 KB)

## Data Availability

The datasets used are available from the corresponding author upon reasonable request.
