# Supplementary material for: Optimisation of Pharmaceutical Cocrystal Dissolution Performance through a Synergistic Precipitation Inhibition
Source: Pharm Res. 2023 May 15;40(8):2051–69. doi: 10.1007/s11095-023-03532-x (PMC10447287; doi:10.1007/s11095-023-03532-x)
Supplement: Supplementary file 1 — Supplementary file1 (DOCX 2797 KB) [file 11095_2023_3532_MOESM1_ESM.docx]

**Supporting materials**

**Optimisation of Pharmaceutical Cocrystal Dissolution Performance through a** **Synergistic Precipitation Inhibition**

Kejing Shi and Mingzhong Li^[[1]](#footnote-2)^*

School of Pharmacy, De Montfort University, Leicester LE1 9BH, U.K.

Table S1: Phase solubility diagram study.

Note: 1) total amount of FFA and NIC in each vial represented 10% w/w of the total sample weight; 2) total volume of solvent in each vial was 10 mL, representing 90% w/w of the total sample weight.

1. FFA and NIC in DDW

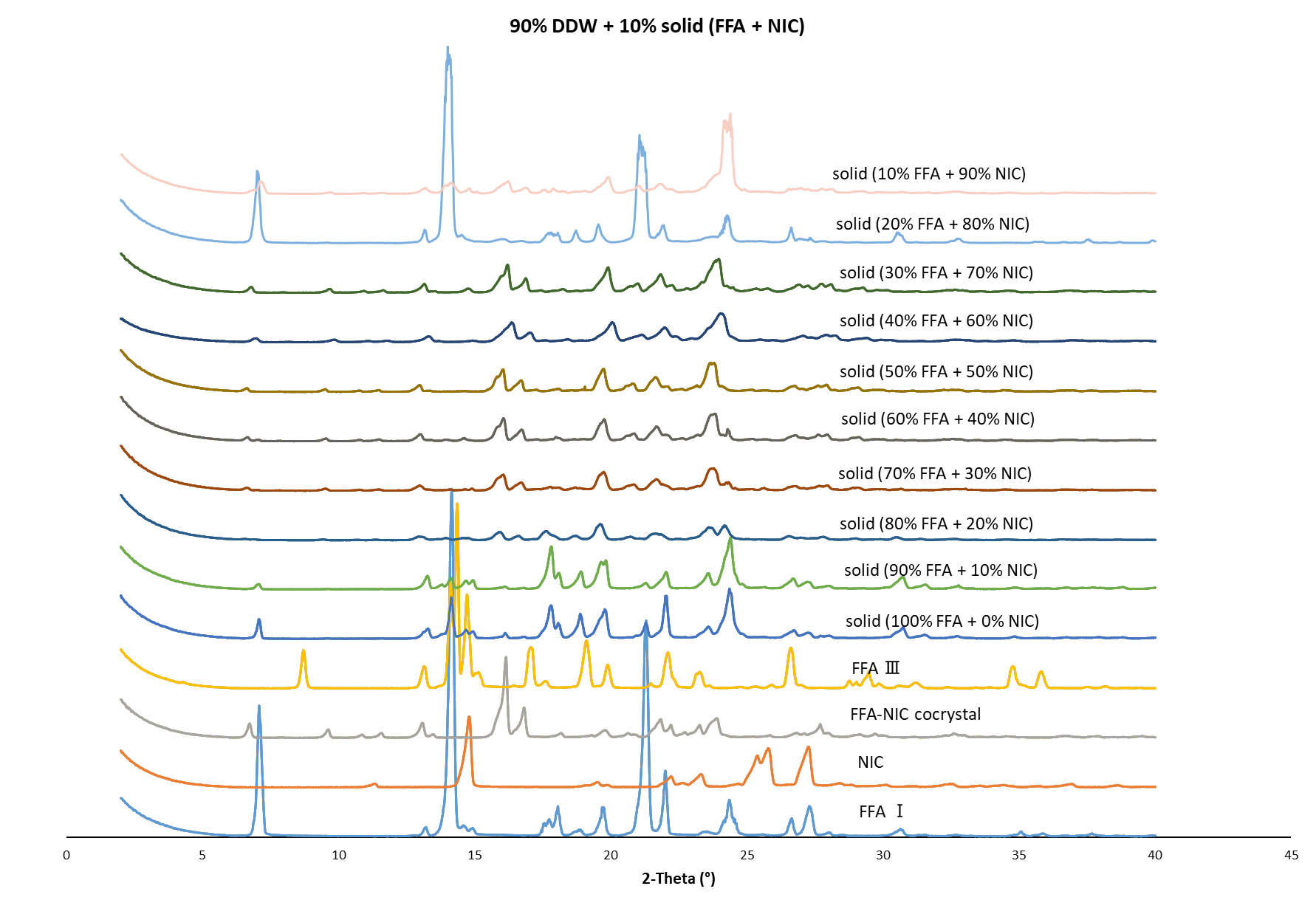


(2) FFA and NIC in PVP-VA (0.2 mg/mL) predissovled DDW

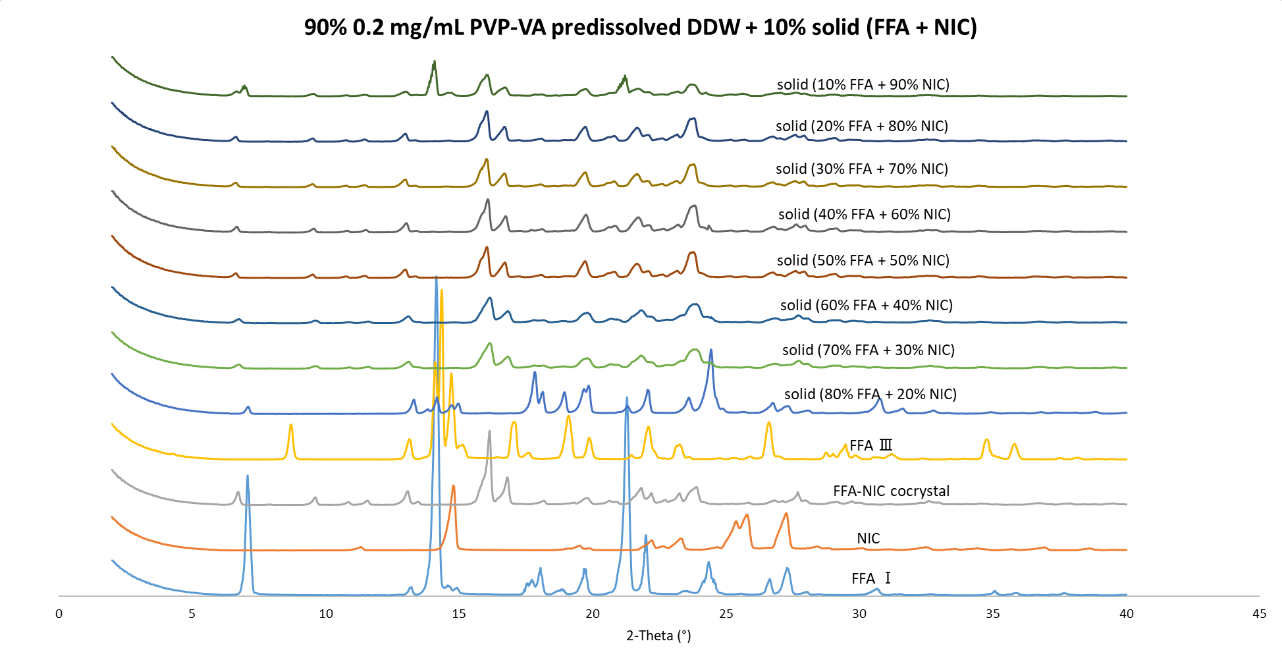


(3) FFA and NIC in PEG (1 mg/mL) predissovled DDW

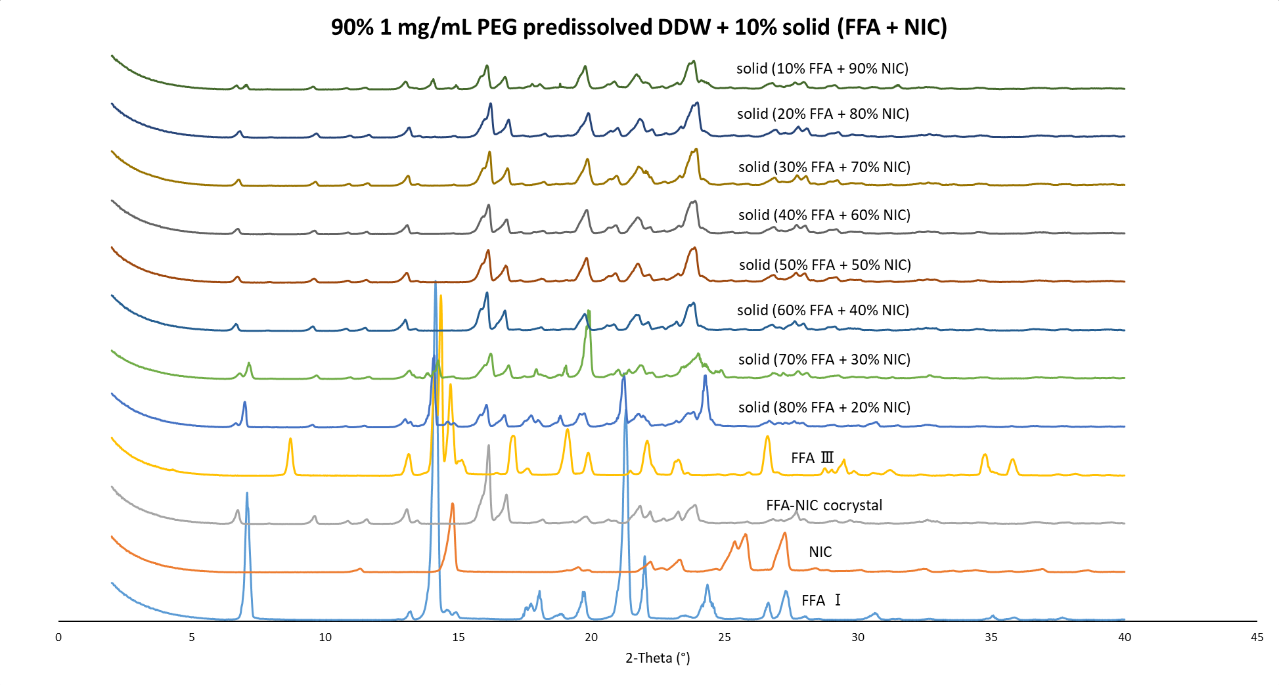


(4) FFA and NIC in SLP (0.2 mg/mL) predissovled DDW

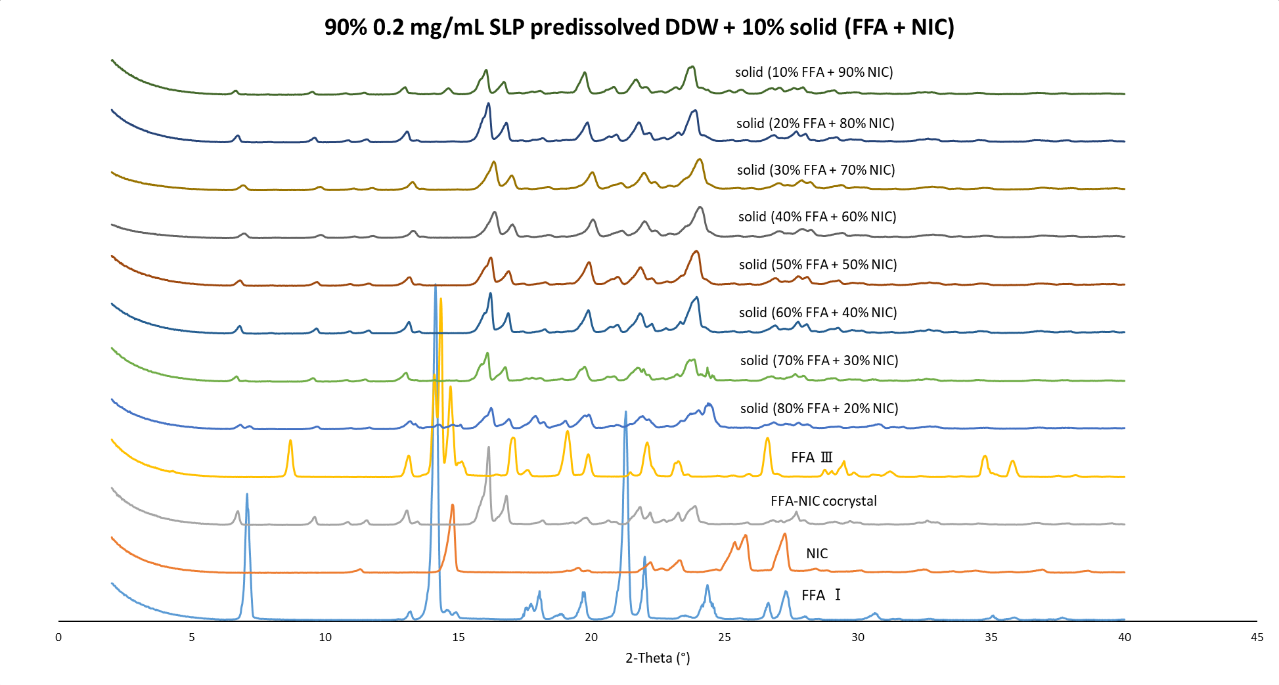


(5) FFA and NIC in PEG (1 mg/mL) + PVP-VA (0.2 mg/mL) predissovled DDW

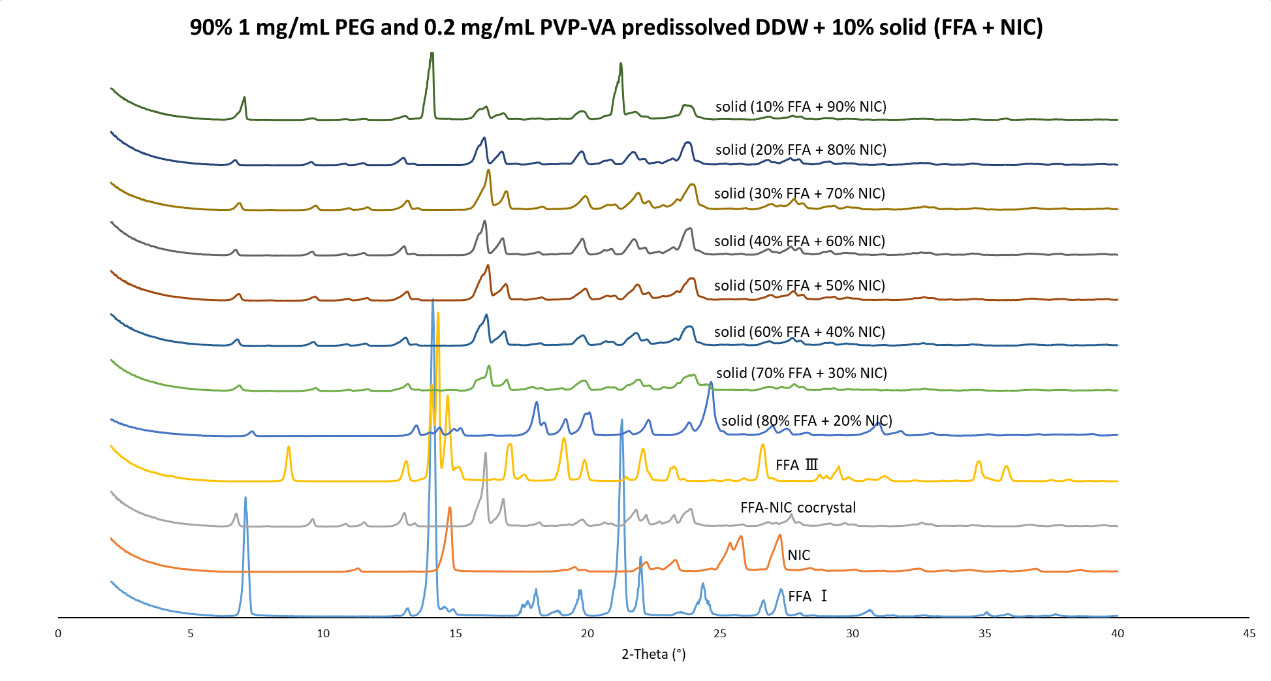


(6) FFA and NIC in SLP (0.2 mg/mL) + PVP-VA (0.2 mg/mL) predissovled DDW

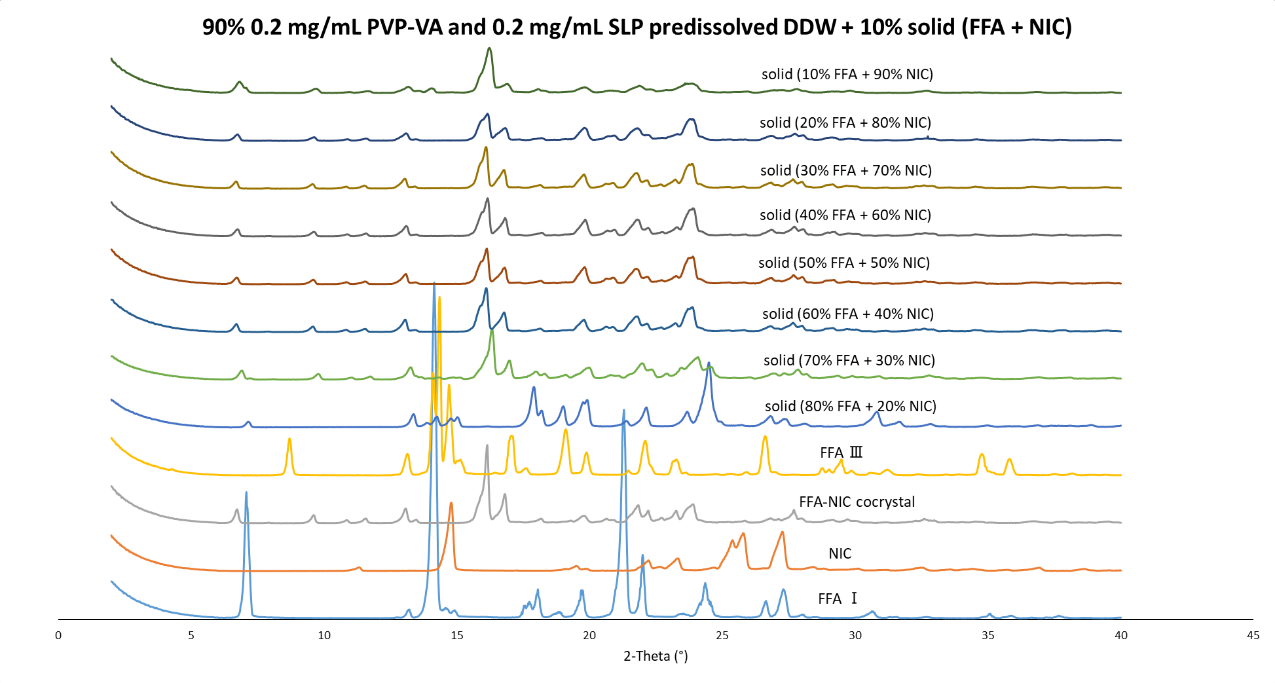


(7) FFA and NIC in PBS pH 4.5

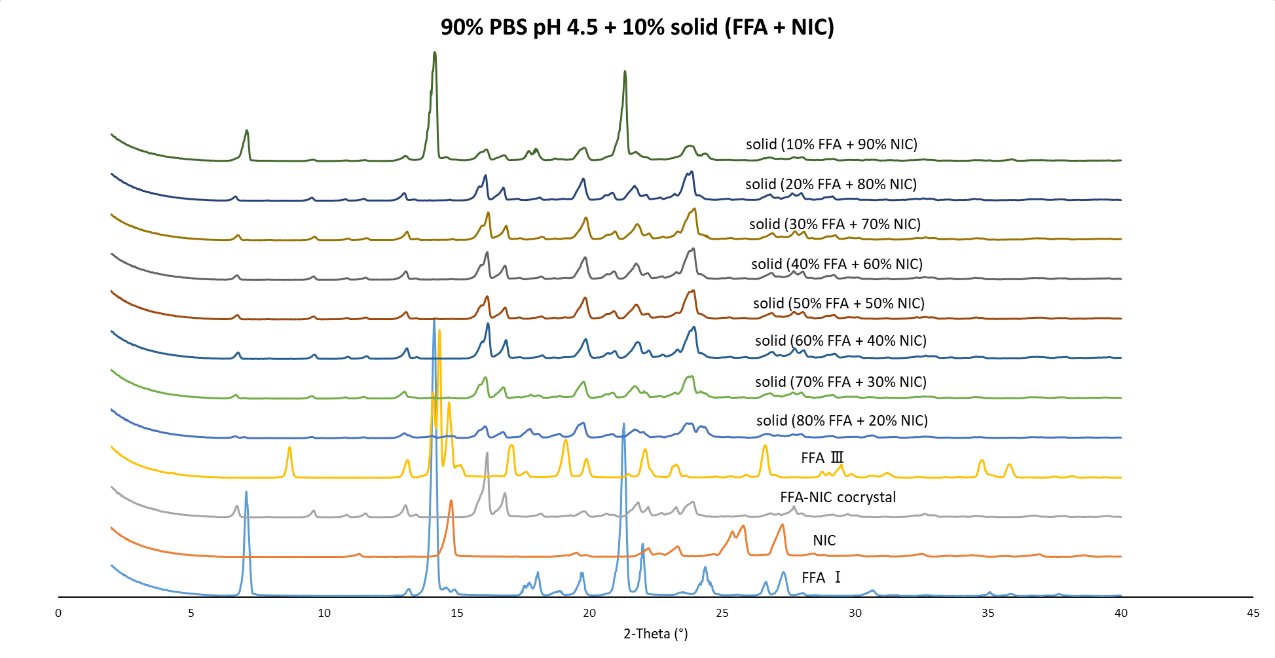


(8) FFA and NIC in PVP-VA (0.2 mg/mL) PBS pH 4.5

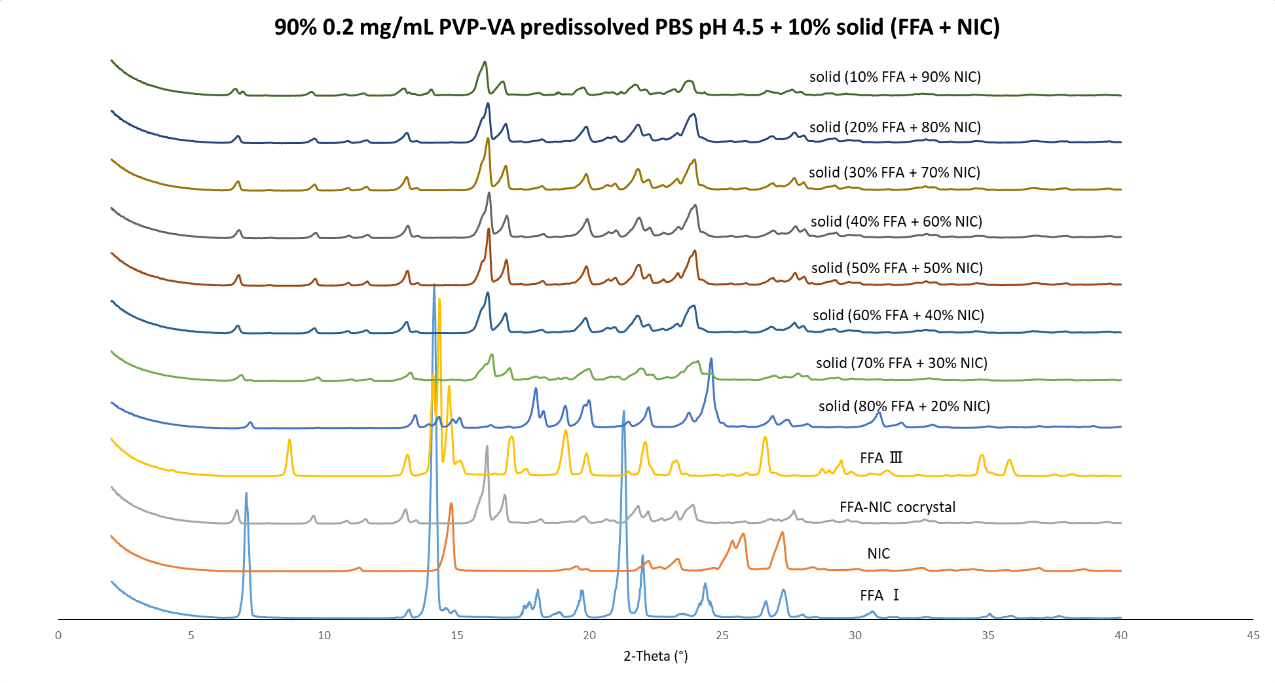


(9) FFA and NIC in PEG (1 mg/mL) PBS pH 4.5

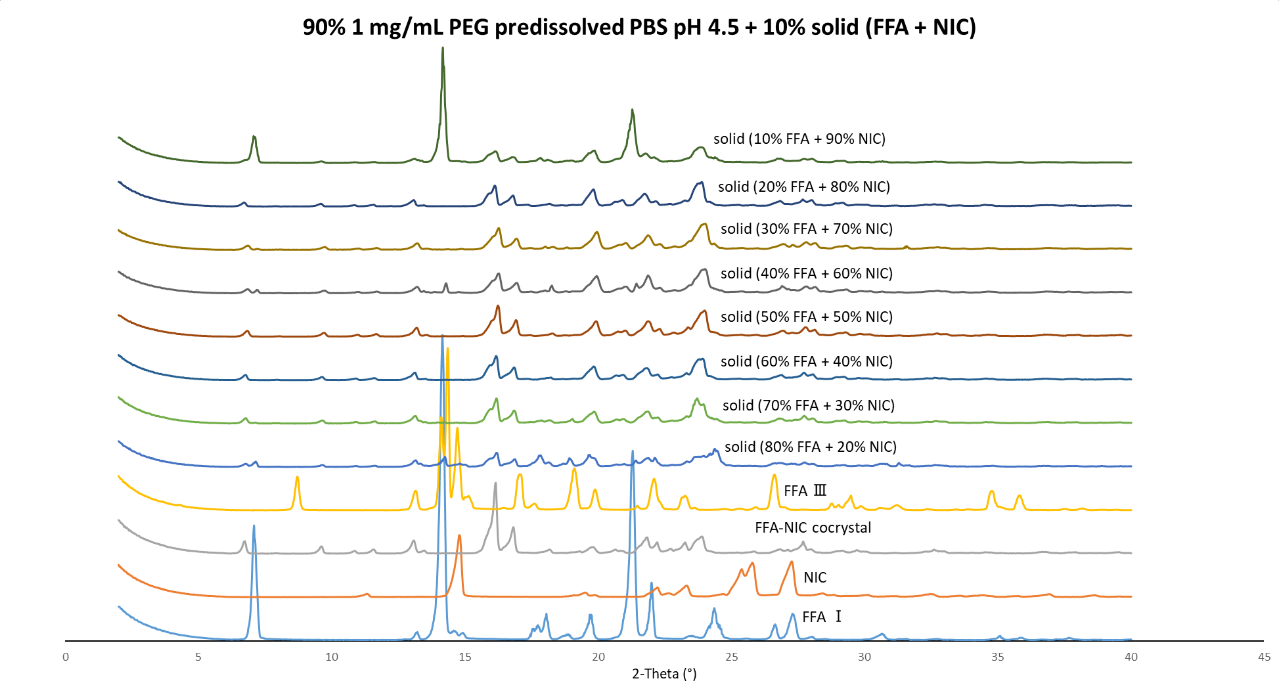


(10) FFA and NIC in SLP (0.2 mg/mL) PBS pH 4.5

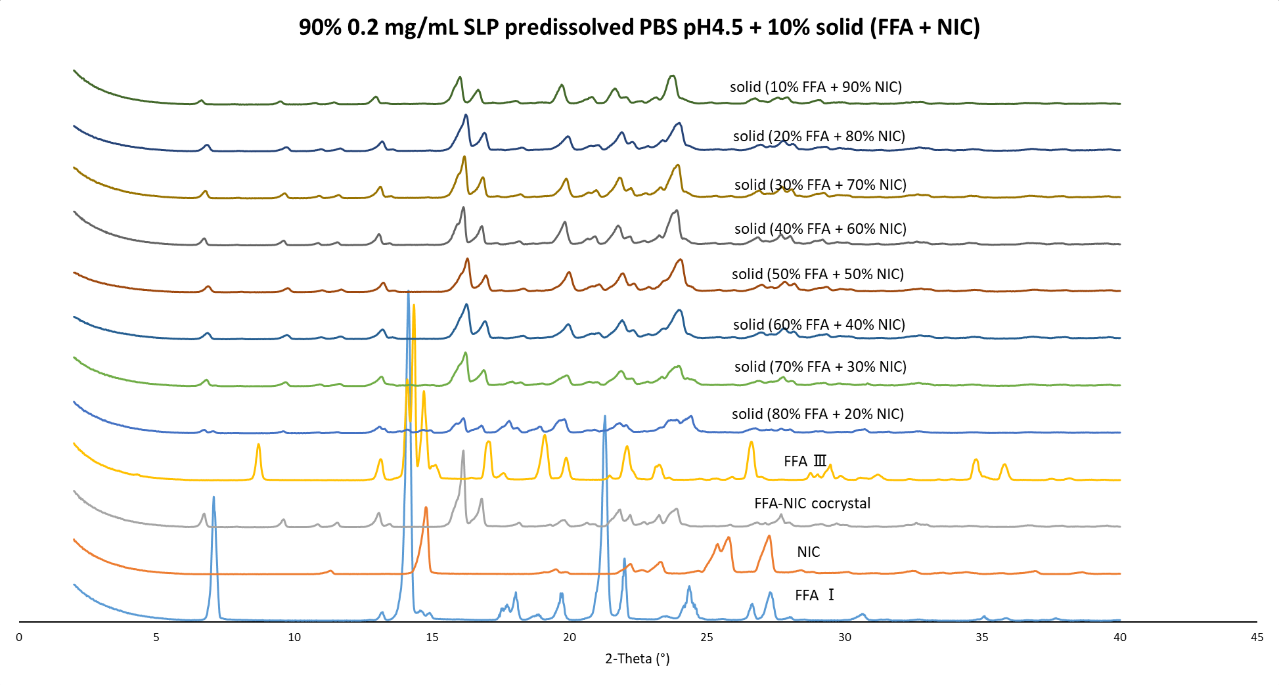


(11) FFA and NIC in PEG (1 mg/mL) + PVP-VA (0.2 mg/mL) predissovled PBS pH 4.5

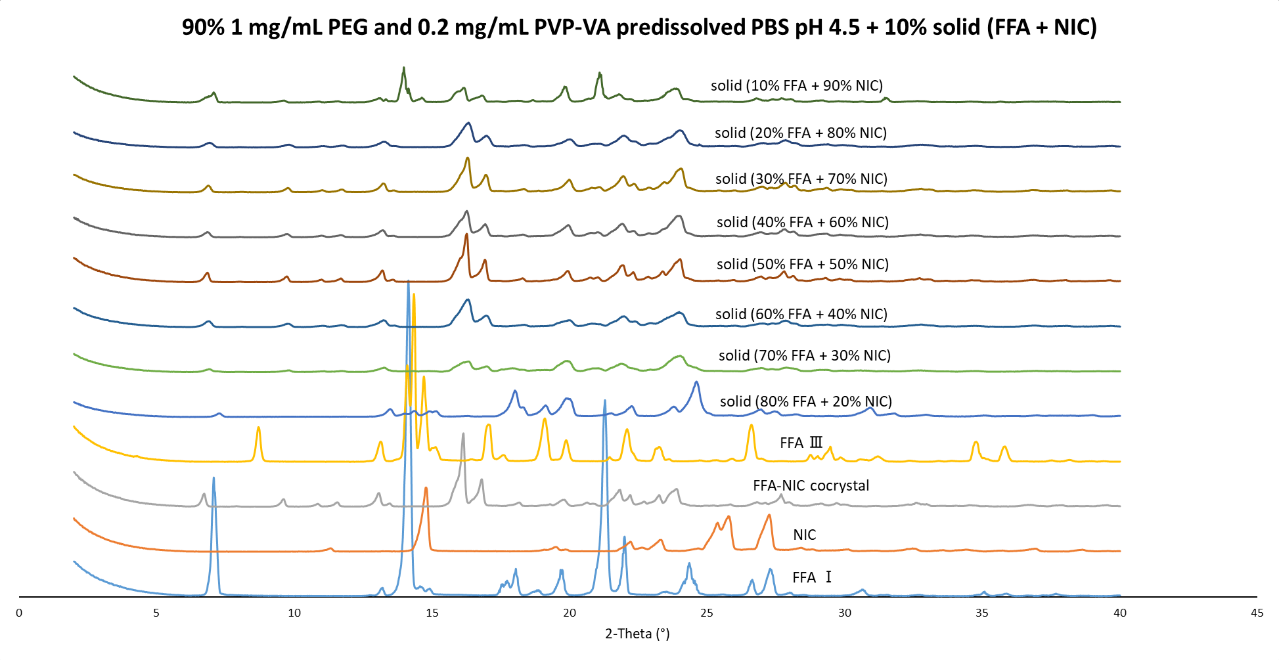


(12) FFA and NIC in SLP (0.2 mg/mL) + PVP-VA (0.2 mg/mL) predissovled PBS pH 4.5

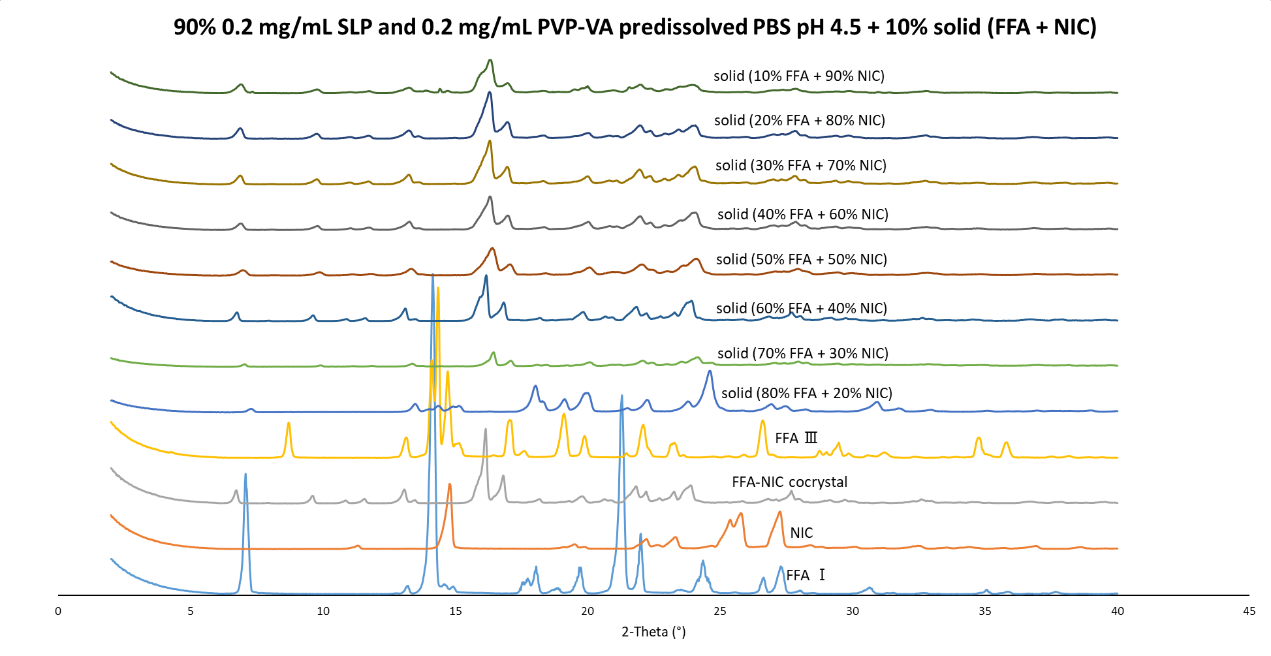


Table S2: The measured values of solubility of FFA (µg/mL)

| FFA solubility (μg/mL) | | | |
| --- | --- | --- | --- |
| solvents | DDW | PBS pH 4.5 | PBS pH 6.8 |
| absence of polymer | 10.06±0.79 | 18.27±2.39 | 741.15±6.27 |
| predissolved PVP-VA (0.2 mg/mL) | 12.04±1.40 | 10.98±1.32 | - |
| predissolved PEG (1 mg/mL) | 17.78±1.72 | 9.01±0.26 | - |
| predissolved SLP (0.2 mg/mL) | 110.54±16.22 | 41.89±2.50 | - |
| predissolved PEG (1 mg/mL) + PVP-VA (0.2 mg/mL) | 20.89±5.35 | 11.44±0.22 | - |
| predissolved SLP (0.2 mg/mL) + PVP-VA (0.2 mg/mL) | 119.27±3.30 | 41.08±4.20 | - |

Figure S1: Particle size distribution comparison of representative FFA, FFA-TP cocrystal and FFA-NIC powders after sieving.


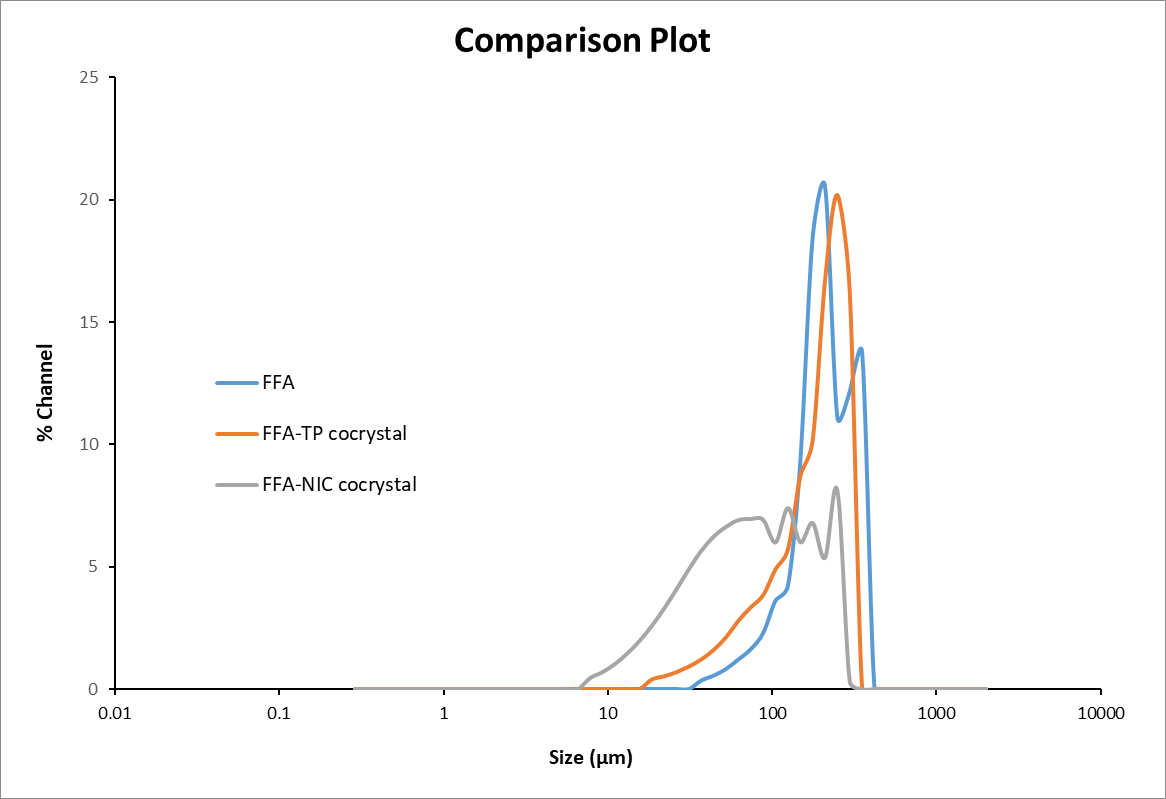


Figure S2: PXRD results of the solid residues collected after the FFA solubility tests in: (a) DDW; (b) PBS pH 4.5.


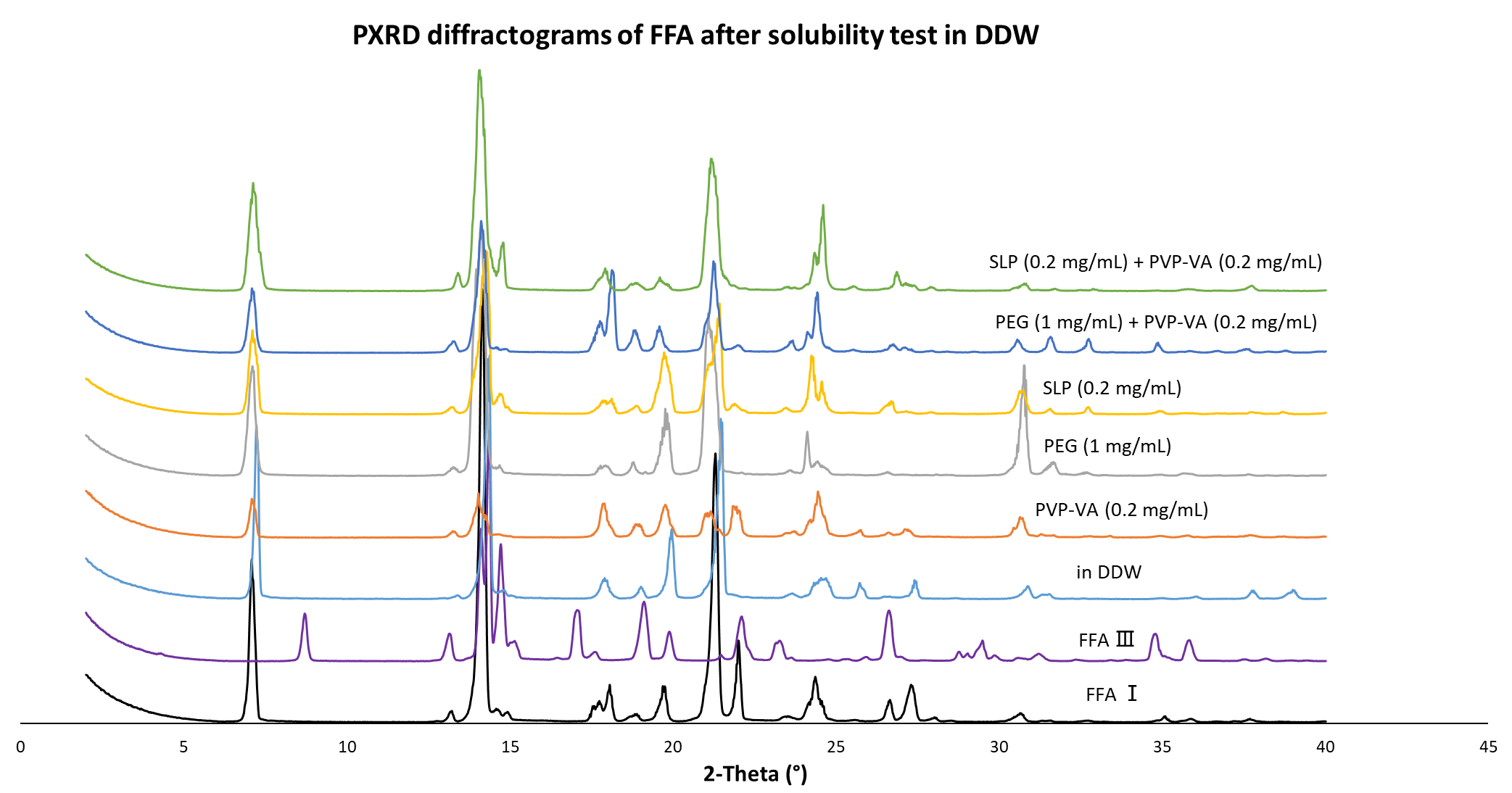


(a)


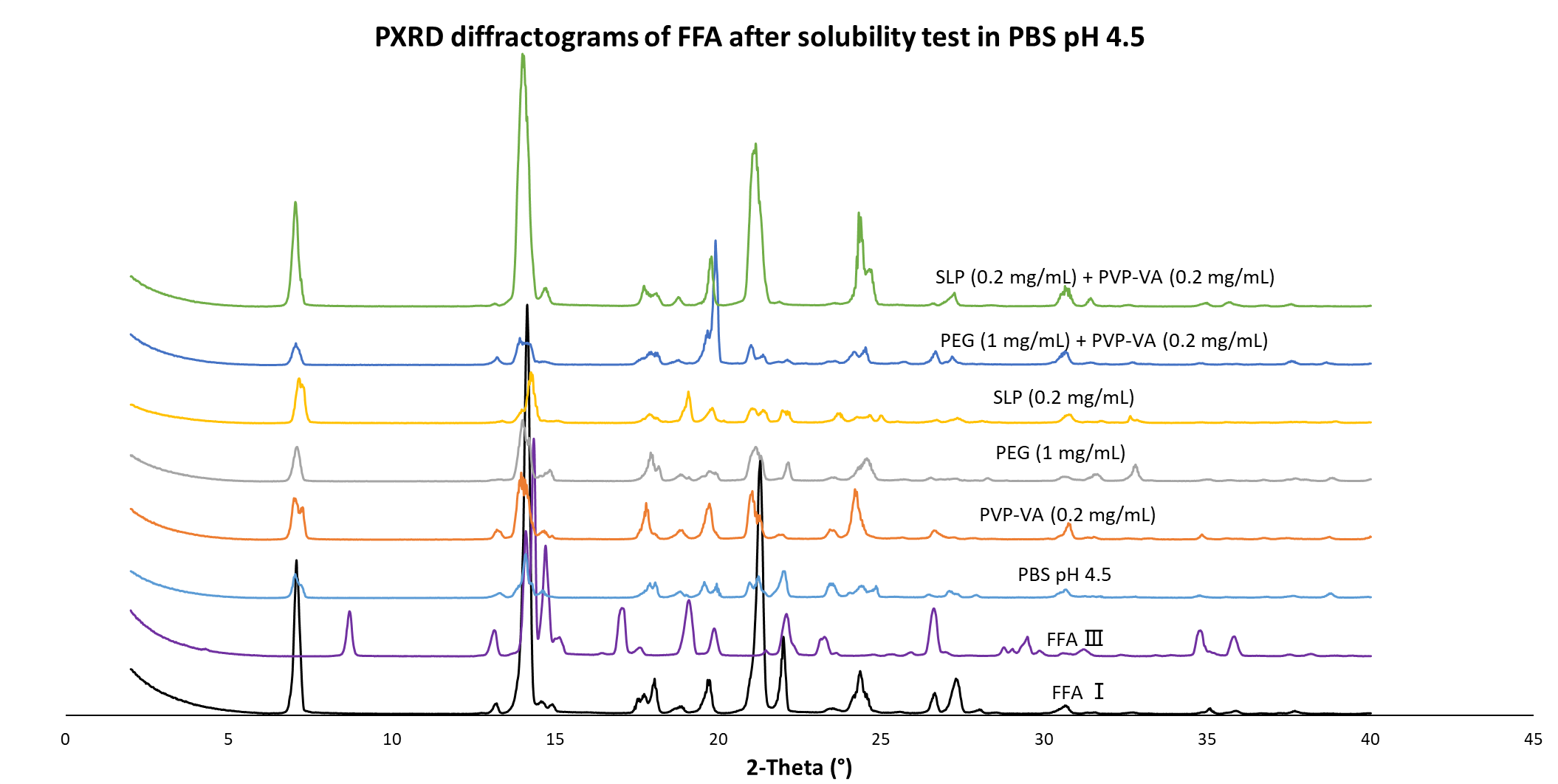


(b)

Figure S3: PSDs of FFA-NIC cocrystal in the absence and presence of a predissolved polymer or a combination in: (a) DDW; (b) PBS pH4.5.

Note: The red dashed line represents the 1:1 stoichiometric composition in solution during FFA-NIC cocrystal dissolution.


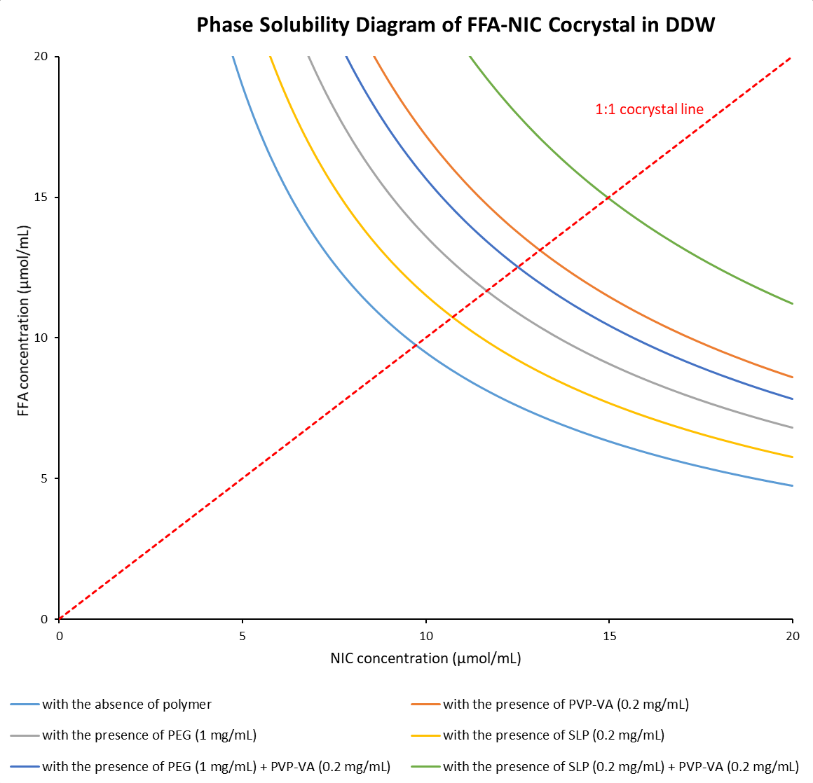


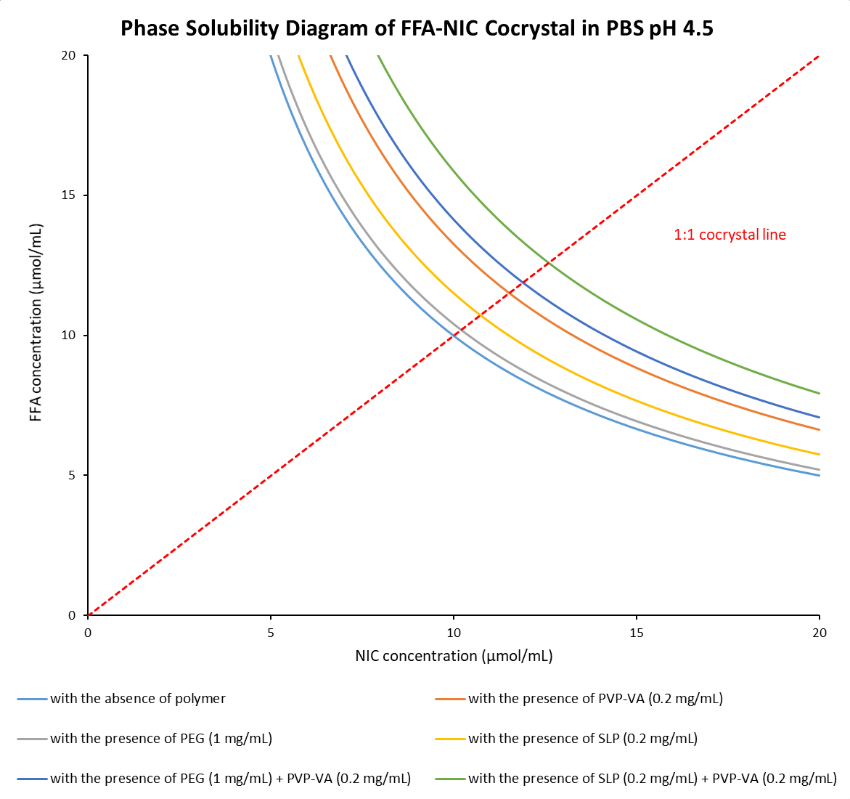


Figure S4: Dissolution performances of the FFA-TP cocrystal powders in DDW in the absence and presence of a predissolved polymer or a combination: a) TP concentration profile; b) dyamic incongruent curve of molar concentrations of FFA vs TP in solution;


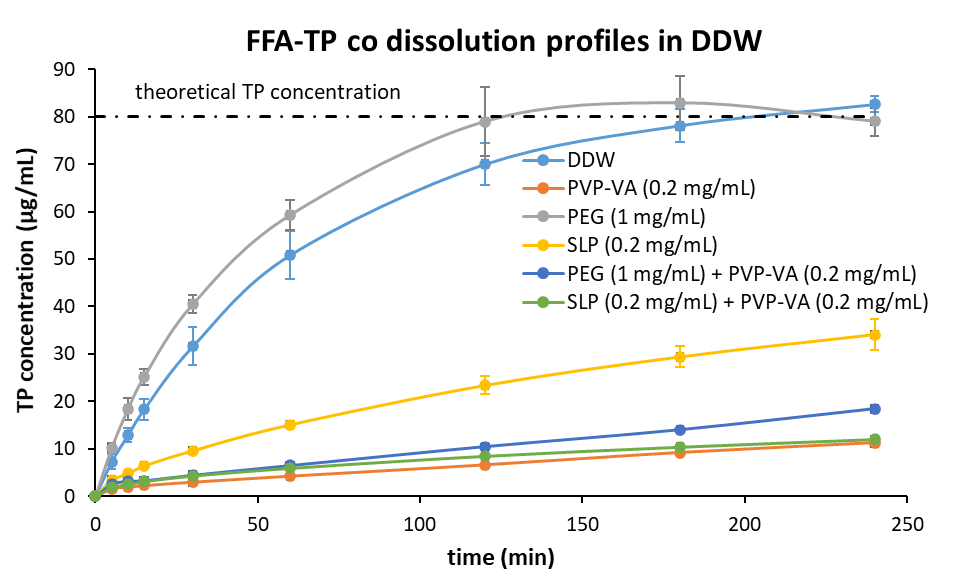


(a)


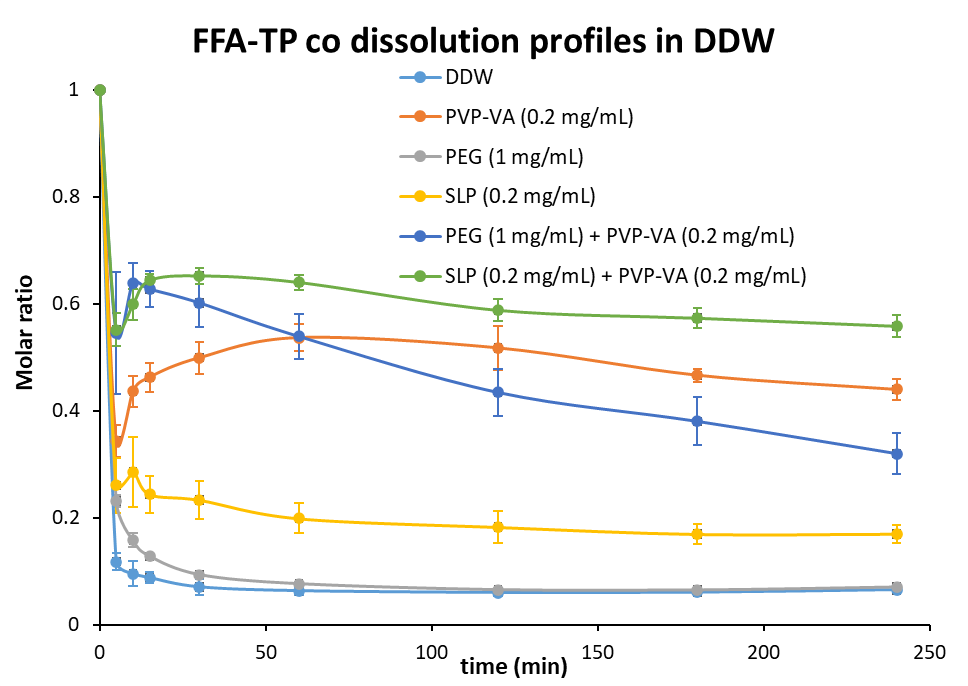


(b)

Figure S5: Trendline plotted according to the solubility data (n = 3) of FFA in various concentrations of SLP predissolved DDW.


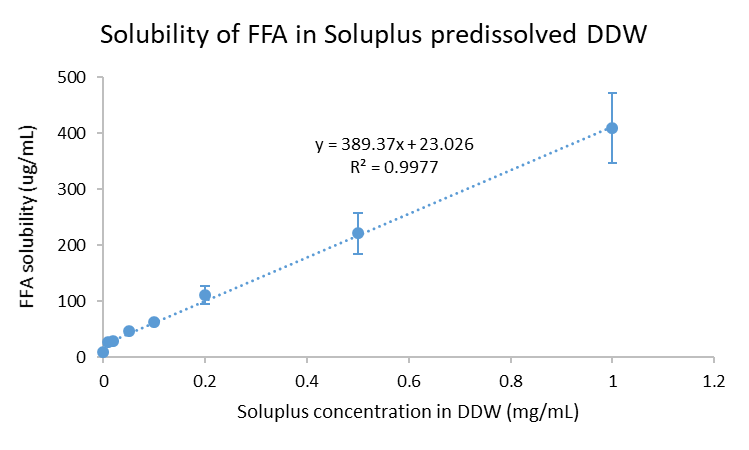


Figure S6: Comparation of dissolution profiles of drug powders in DDW


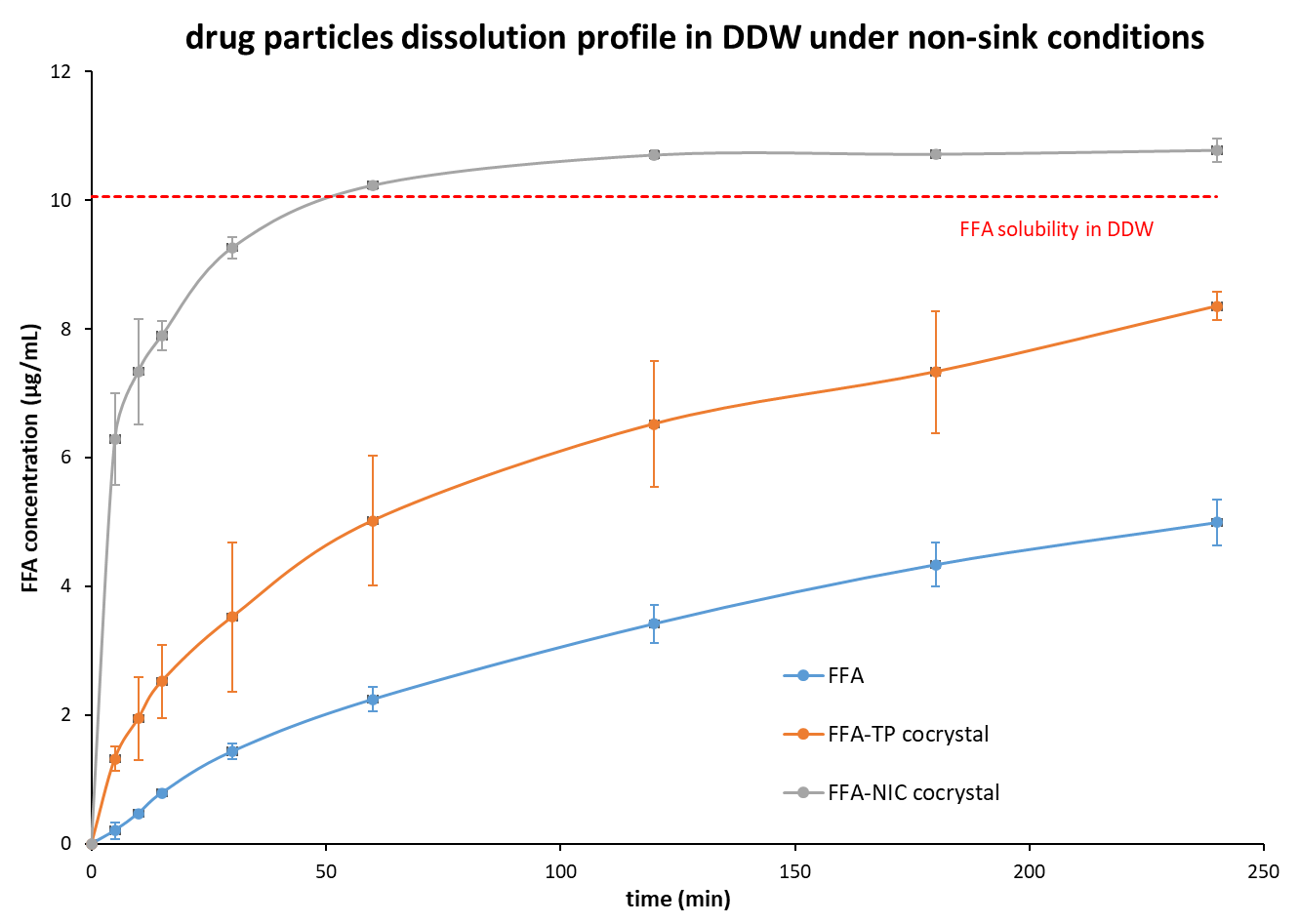


1. * Corresponding author: Phone: +44 (0)116 2577132; Email: mli@dmu.ac.uk [↑](#footnote-ref-2)
